# Metabolic bottlenecks of *Pseudomonas taiwanensis* VLB120 during growth on d-xylose via the Weimberg pathway

**DOI:** 10.1016/j.mec.2024.e00241

**Published:** 2024-06-06

**Authors:** Philipp Nerke, Jonas Korb, Frederick Haala, Georg Hubmann, Stephan Lütz

**Affiliations:** Chair for Bioprocess Engineering, Department of Biochemical and Chemical Engineering, TU Dortmund University, Emil-Figge-Straße 66, 44227, Dortmund, Germany

**Keywords:** Xylose utilization, Renewable feedstocks, Weimberg pathway, *Pseudomonas taiwanensis* VLB120, Xylonolactonase, Xylonate transport

## Abstract

The microbial production of value-added chemicals from renewable feedstocks is an important step towards a sustainable, bio-based economy. Therefore, microbes need to efficiently utilize lignocellulosic biomass and its dominant constituents, such as d-xylose. *Pseudomonas taiwanensis* VLB120 assimilates d-xylose via the five-step Weimberg pathway. However, the knowledge about the metabolic constraints of the Weimberg pathway*,* i.e., its regulation, dynamics, and metabolite fluxes, is limited, which hampers the optimization and implementation of this pathway for bioprocesses. We characterized the Weimberg pathway activity of *P. taiwanensis* VLB120 in terms of biomass growth and the dynamics of pathway intermediates. In batch cultivations, we found excessive accumulation of the intermediates d-xylonolactone and d-xylonate, indicating bottlenecks in d-xylonolactone hydrolysis and d-xylonate uptake. Moreover, the intermediate accumulation was highly dependent on the concentration of d-xylose and the extracellular pH. To encounter the apparent bottlenecks, we identified and overexpressed two genes coding for putative endogenous xylonolactonases PVLB_05820 and PVLB_12345. Compared to the control strain, the overexpression of PVLB_12345 resulted in an increased growth rate and biomass generation of up to 30 % and 100 %, respectively. Next, d-xylonate accumulation was decreased by overexpressing two newly identified d-xylonate transporter genes, PVLB_18545 and *gntP* (PVLB_13665). Finally, we combined xylonolactonase overexpression with enhanced uptake of d-xylonate by knocking out the *gntP* repressor gene *gntR* (PVLB_13655) and increased the growth rate and biomass yield by 50 % and 24 % in stirred-tank bioreactors, respectively. Our study contributes to the fundamental knowledge of the Weimberg pathway in pseudomonads and demonstrates how to encounter the metabolic bottlenecks of the Weimberg pathway to advance strain developments and cell factory design for bioprocesses on renewable feedstocks.

## Highlights

• d-xylonolactone hydrolysis and d-xylonate uptake present metabolic bottlenecks.
• The extracellular pH strongly influences the growth and accumulation of d-xylonolactone.
• Overexpression of a putative xylonolactonase leads to reduced accumulation of d-xylonolactone and optimized growth.
• Two novel d-xylonate transporters improve growth and d-xylonate uptake.
• Improved growth rate and yield based on the renewable carbon source d-xylose.

## 1 Introduction

The microbial production of value-added chemicals from renewable feedstocks plays an important role in the structural transformation from a petrochemical industry to a sustainable bioeconomy (Intasian et al., 2021). First-generation feedstocks are mostly based on d-glucose and are derived, for example, from sugarcane or corn, which are also used in human nutrition. In contrast to that, second-generation feedstocks are derived from non-edible lignocellulosic biomass and thus avoid the direct competition with the agricultural food production (Bardhan et al., 2015). The most abundant sugar building block in lignocellulose after d-glucose is the pentose d-xylose. For example, d-xylose constitutes about 15 % of the total dry mass in corn stover and 23 % in bagasse fibers (Lee, 1997). Hence, the efficient utilization of d-xylose is of great interest.

The ability to catabolize d-xylose differs greatly among microorganisms and d-xylose is consumed as a carbon and energy source in a variety of pathways, namely the isomerase pathway, the xylose reductase/xylitol dehydrogenase pathway, the Dahms pathway and the Weimberg pathway (Kim and Woo, 2018; Narisetty et al., 2022; Ramos et al., 2018; Zhao et al., 2020). An additional non-phosphorylative pathway was discovered only recently (Watanabe et al., 2019). Moreover, artificial pathways are developed, such as the synthetic xylulose-1P pathway (Cam et al., 2016). Among the metabolic pathways for the utilization of d-xylose, the Weimberg pathway has some unique features that are advantageous for certain bioprocesses. Firstly, the five-reaction step pathway efficiently converts d-xylose to α-ketoglutarate without carbon loss in the form of CO_2_. Secondly, no ATP is consumed in the pathway, making it more energy efficient. Thirdly, the pathway intermediates do not interfere with other major pathways, which results in a short and linear pathway up to α-ketoglutarate. Fourthly, all reactions are thermodynamically favorable as they have negative standard Gibbs free energy changes (Shen et al., 2020). Lastly, the Weimberg pathway offers direct access to produce and replenish tricarboxylic acid (TCA) cycle-derived metabolites. Due to its direct link to the TCA cycle via α-ketoglutarate, the Weimberg pathway enables, for instance, short synthesis routes for α-ketoglutarate (Brüsseler et al., 2019), succinate (Tenhaef et al., 2021), glutaric acid (Wang et al., 2018) and mesaconic acid (Bai et al., 2016). Fueling of TCA cycle intermediates by co-utilization of d-xylose via the Weimberg pathway was shown to help overcoming glutamate auxotrophy in an itaconate-producing *E. coli* strain (Lu et al., 2021) and to provide increased amounts of α-ketoglutarate for biotransformations with α-ketoglutarate-dependent dioxygenases (Wei et al., 2022).

Pseudomonads represent valuable hosts for cell factories due to their versatile metabolism, stress- and solvent tolerance (Bitzenhofer et al., 2021; Schwanemann et al., 2020). Moreover, they can be readily engineered by contemporary molecular biology methods (Nikel and de Lorenzo, 2021). *Pseudomonas taiwanensis* VLB120 is a promising chassis organism with high solvent tolerance and has been efficiently engineered, for example, for styrene epoxidation (Park et al., 2007; Volmer et al., 2017), the synthesis of adipic acid and 6-hydroxy-hexanoic acid from cyclohexane (Bretschneider et al., 2022; Schäfer et al., 2020) or the synthesis of 4-hydroxybenzoate (Lenzen et al., 2019). The strain has potential for the valorization of lignocellulosic biomass, as it is tolerant towards lignocellulosic biomass-derived inhibitors, such as vanillin or furfural (Wordofa and Kristensen, 2018). Moreover, *P. taiwanensis* VLB120 natively catabolizes d-xylose via the Weimberg pathway (Köhler et al., 2015). However, the knowledge of the regulation, dynamics, and transport of the Weimberg pathway metabolites is still fragmented, although the pathway has been discovered in Pseudomonas species over half a century ago (Lockwood and Nelson, 1946; Weimberg, 1961).

**Fig. 1:**
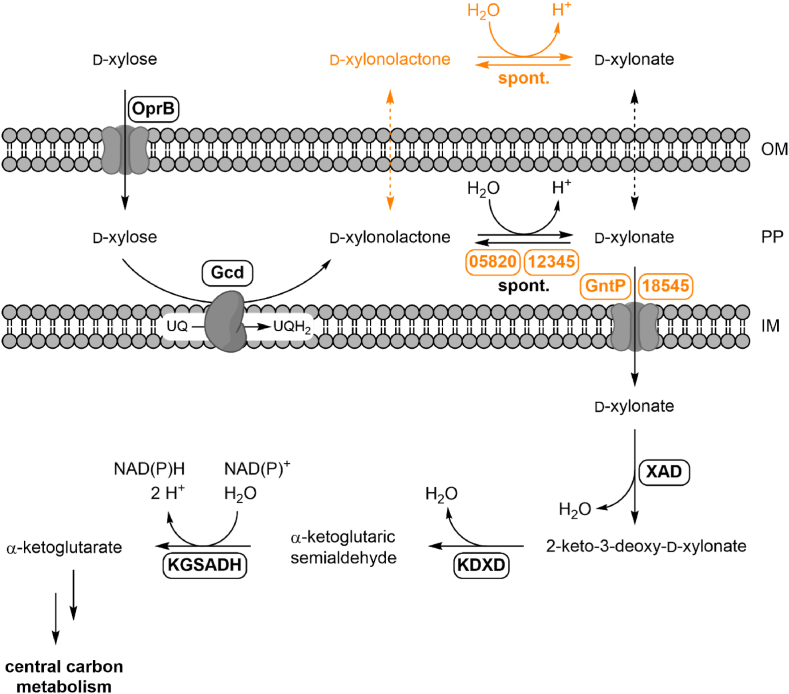
**Schematic representation of the Weimberg pathway from *P. taiwanensis* VLB120.** New insights from this publication are highlighted in orange. d-Xylose is hypothesized to be taken up into the periplasm by porin B (OprB). The periplasmic pyrroloquinoline quinone-dependent glucose dehydrogenase (Gcd, PVLB_05240) performs the conversion to d-xylonolactone. Electrons are transferred to ubiquinone (UQ), which is reduced to ubiquinol (UQH_2_). D-xylonolactone is hydrolyzed spontaneously or by a xylonolactonase (XLA, PVLB_05820/PVLB_12345) to d-xylonate. d-Xylonate is taken up into the cell by the two transporters GntP (PVLB_13665) and PVLB_18545. Xylonate dehydratase (XAD, PVLB_18565) performs the conversion to 2-keto-3-deoxy-d-xylonate and 2-keto-3-deoxy-d-xylonate dehydratase (KDXD, PVLB_18560) to α-ketoglutaric semialdehyde. Conversion to α-ketoglutarate is performed by α-ketoglutarate semialdehyde dehydrogenase (KGSADH, PVLB_11380/PVLB_18510/PVLB_18550). OM (outer membrane), PP (periplasm), IM (inner membrane). The numbers in the figure represent locus tags without PVLB prefix. (For interpretation of the references to colour in this figure legend, the reader is referred to the Web version of this article.)

The most studied genes and enzymes of the Weimberg pathway originate from an operon of *Caulobacter crescentus* (Stephens et al., 2007). After the discovery of the operon, the pathway has been recombinantly introduced in several industrially relevant strains such as *Pseudomonas putida* (Meijnen et al., 2009), *E. coli* (Rossoni et al., 2018; Tai et al., 2016), *Corynebacterium glutamicum* (Radek et al., 2014), *Bacillus subtilis* (Halmschlag et al., 2020) and *Saccharomyces cerevisiae* (Borgström et al., 2019; Wasserstrom et al., 2018). However, these recombinant strains often suffer from low growth rates and/or accumulation of the intermediate d-xylonate, which hampers their use as production host in bioprocesses (Bañares et al., 2021; Bator et al., 2019; Lim et al., 2021; Rossoni et al., 2018). Shen et al. (2020) investigated the Weimberg pathway as an enzymatic cascade *in vitro* and identified potential bottlenecks to be the recycling of NAD^+^, balanced activities of the dehydratases and insufficient autohydrolysis of d-xylonolactone at higher pathway fluxes. Their findings explain the encountered issues *in vivo* to some degree, however NAD^+^ recycling is not to be expected as a bottleneck in aerobically growing cells. In contrast, the issue of substrate uptake and metabolite leakage are inherently not considered in an *in vitro* approach. Moreover, in contrast to *Caulobacter crescentus*, the natural Weimberg pathway in pseudomonads is divided into different compartments, which has profound influence on its metabolic activity (Fig. 1). d-Xylose is taken up into the periplasm, presumably by OprB (Trias et al., 1988) and the periplasmic pyrroloquinoline quinone-dependent glucose dehydrogenase Gcd performs the conversion to d-xylonolactone. Moreover, this reaction couples the Weimberg pathway to aerobic respiration via the generation of ubiquinol (Zannoni, 2004). In the periplasm, d-xylonolactone is hydrolyzed to d-xylonate spontaneously or by a xylonolactonase. This reaction is subject to a pH-dependent thermodynamic equilibrium (Hummel et al., 2010). d-Xylonate and in some cases d-xylonolactone have been found to accumulate in the medium, which indicates a yet unknown bottleneck (Bator et al., 2019; Buchert and Viikari, 1988; Köhler et al., 2015; Weimberg, 1961). d-Xylonate uptake and efflux mechanisms remain largely unknown and are mainly inferred from gluconate transport. Until now, only the 2-ketogluconate transporter KguT has been recently identified as a xylonate transporter in *P. putida* KT2440 (Lim et al., 2021). In contrast to other pseudomonads, such as *P. putida* KT2440, *P. taiwanensis* VLB120 harbors all Weimberg pathway genes and is naturally able to catabolize d-xylose via the Weimberg pathway (Köhler et al., 2015). Additionally, a dependency of the growth rate on the d-xylose concentration has been indicated for the strain (Wordofa and Kristensen, 2018). Moreover, the growth rate increased notably, when the strain was grown on d-xylonate as the only carbon source instead of d-xylose (Köhler et al., 2015). Hence, the Weimberg pathway in *Pseudomonas* species requires special attention to generate a suitable catabolic pathway for bioprocesses.

In this study, we characterize in depth the physiological response and growth of *P. taiwanensis* VLB120 on d-xylose via the Weimberg pathway, applying various nutritional conditions. We found that, aside from d-xylonate, d-xylonolactone accumulated in various batch cultivations and different bioreactors. Moreover, the intermediate accumulation is highly dependent on the concentration of d-xylose and the extracellular pH. To address the apparent *in vivo* bottlenecks, we applied rational metabolic engineering of the Weimberg pathway for optimizing the growth of *P. taiwanensis* VLB120 on d-xylose. Moreover, we identified two previously unknown d-xylonate transporters. To use the entire capacity of d-xylonate transport, we deregulated the transport by deleting the GntP-controlling repressor GntR. Combining overexpression of a xylonolactonase and the knockout of *gntR*, we reduced the intermediate accumulation and improved the growth rate and biomass yield of *P. taiwanensis* VLB120 on d-xylose.

## 2 Material and methods

### 2.1 Chemicals and culture media

d-xylose was purchased from Sigma-Aldrich Chemie GmbH (St. Louis, USA), and d-xylonolactone was purchased from Carbosynth Ltd. (Compton, UK). All other chemicals were acquired from Carl Roth GmbH + Co. KG (Karlsruhe, Germany) and Merck KGaA (Darmstadt, Germany). Oligonucleotides for cloning were also obtained from Sigma-Aldrich. Bacterial strains in this study were cultivated in lysogeny broth (LB) medium or M9 medium (Sambrook and Russell, 2001) with varying amounts of d-xylose, NH_4_Cl, and different pH values depending on the scientific question. The exact compositions are listed in the supplementary information (Table S1). The antibiotics streptomycin (100 μg mL^−1^, Sm^100^), kanamycin (50 μg mL^−1^, Km^50^), and gentamicin (25 μg mL^−1^, Gm^25^) were supplemented to the growth media as needed.

### 2.2 Bacterial strains and plasmids

Bacterial strains and plasmids from this study are listed in Table S2 and Table S3. *Escherichia coli* DH5α was used for plasmid construction and propagation. *P. taiwanensis* VLB120 strains were used for physiological characterization. *P. taiwanensis* VLB120 contains a megaplasmid pSTY, which is not essential for survival. As the megaplasmid easily gets lost during genetic manipulations (Wynands et al., 2019), we used *Pseudomonas taiwanensis* VLB120ΔC, which harbors a streptomycin resistance on the megaplasmid. The additional marker ensured retention of the megaplasmid throughout all the experiments. *Pseudomonas putida* KT2440 was used as a donor for the 2-ketogluconate transporter gene *kguT*. The plasmids from this study were based on the plasmid pCom10lac (Lindmeyer, 2016) and constructed as described in 2.4.

### 2.3 Bacterial cultivation

*P. taiwanensis* VLB120 strains were streaked on LB agar plates from cryogenic stock cultures and cultivated overnight at 30 °C. A single colony was used to inoculate a 2 mL LB preculture in 12 mL cultivation tubes. The preculture was cultivated in an orbital shaker for 8 h at 30 °C and 200 rpm (2.5 cm amplitude). After that, the LB preculture was used to inoculate a 25 mL M9 preculture at 1 % (v/v) culture volume in baffled 250 mL Erlenmeyer flasks (liquid:gas phase ratio 1:9) and cultivated overnight (30 °C, 200 rpm, 2.5 cm amplitude). The OD_450_ of the precultures was determined, to inoculate the M9 main cultures. The amount needed to inoculate the desired volume of the main culture with an OD_450_ of 0.2 was transferred to separate sterile reaction tubes and centrifuged. The cell pellet was resuspended in M9 medium and added to the main culture. Standard main cultures were cultivated in baffled 500 mL Erlenmeyer flasks (liquid:gas phase ratio 1:9) at 30 °C and 150 rpm (2.5 cm amplitude). The strains containing gene knockouts for the identification of the d-xylonate transporters, only one 10 mL LB preculture was inoculated in 100 mL baffled Erlenmeyer flasks from respective agar plates and cultivated overnight (30 °C, 200 rpm, 2.5 cm amplitude).

#### 2.3.1 Microbioreactor batch cultivations

Parallel microbioreactor cultivations were performed in a BioLector I (m2p-labs, Baesweiler, Germany). The cultivations were started with an OD_450_ of 0.2. Experiments were conducted in 48-well microplates (FlowerPlate, MTP-48-B) at 1 mL scale, 30 °C, 1200 rpm, and humidity control (85 %). In the growth experiments with transporter gene overexpression, 0.1 mM IPTG was added 12 h after inoculation. During growth, the backscatter at 620 nm was measured to monitor growth characteristics. After the cultivation, the final OD_450_ was measured, samples were taken, and centrifuged at 21,100×*g* and 4 °C. Afterwards, the supernatant was transferred to a new reaction tube and stored at −20 °C until further analysis.

#### 2.3.2 Cultivation in Erlenmeyer flasks with growth monitoring

Cultivations in Erlenmeyer flasks with automated backscatter measurement at 521 nm were performed in a Multitron standard shaker with 2.5 cm amplitude (Infors HT, Bottmingen, Switzerland) equipped with a cell growth quantifier (CGQ) system (Aquila Biolabs, Baesweiler, Germany). As knockout strains might have lost their ability to grow on d-xylose as a substrate, precultures were incubated in LB medium as described above. After harvesting the cells, the 25 mL main culture was started in M9 medium at an OD_450_ of 0.2. The cultures were incubated at 30 °C and 200 rpm for 72 h. During the complementation experiments of the d-xylonate transporter genes, IPTG (0.1 mM) was added in all cultivation steps to ensure a constant expression of the investigated genes. After cultivation, the final OD_450_ was measured, samples were taken, and centrifuged at 11,000×*g* and 4 °C. The supernatant was transferred to a new reaction tube and stored at −20 °C until further analysis.

#### 2.3.3 Cultivation in stirred-tank bioreactors

The stirred-tank bioreactor experiments were conducted using a DASbox parallel bioreactor system (Eppendorf SE, Hamburg, Germany). Precultures in LB medium and M9 medium were cultivated as described above and 10 mL inoculum were used to inoculate the reactors to a liquid volume of 200 mL with an initial OD_450_ of 0.2. The cultivations were carried out at 30 °C, 1000 rpm (Rushton type impeller, 30 mm diameter), and an air volume flow rate of 3 L h^−1^ (0.25 vvm). When needed, the pH was maintained at various constant values utilizing 1 M NaOH and 1 M H_3_PO_4_. During cultivation, dissolved oxygen (DO), gas flow, exhaust gas composition, pH, and temperature were measured. Culture broth samples were withdrawn from the bioreactor for measurement of the OD_450_ and for analysis of extracellular metabolites. Culture broth samples were centrifuged at 21,100×*g* and 4 °C to obtain the supernatant for metabolite analysis. The supernatant was transferred to a new reaction tube and stored at −20 °C until further analysis.

The growth rate μ and biomass yield $Y_{X/S}$ of the different strains were calculated using the time course data of the exponentially growing phases during the cultivation in the stirred-tank bioreactors. Both parameters were estimated by fitting the concentration time courses to a mathematical model assuming a constant exponential growth rate and biomass yield. The regression and parameter estimation were implemented in OriginPro (2023) (OriginLab Corporation, Northampton, MA, USA), using the Nonlinear Curve Fit analysis and the following explicit nonlinear model of exponential growth

$$X\left(t\right)=X_{0}∙e^{\mu ∙t}$$

$$S\left(t\right)=S_{0}-\frac{1}{Y_{X/S}}∙X_{0}∙\left(e^{\mu ∙t}-1\right)$$

where X is the biomass concentration (unit g_CDW_ L^−1^) and S the amount of d-xylose (unit g L^−1^). The initial concentrations $X_{0}$ and $S_{0}$ were implemented as parameters to be fitted to the initial concentrations of the time courses. The uptake of d-xylose was derived from the growth rate and biomass yield, using the following equation:

$$q_{S}=\frac{\mu }{Y_{X/S}}$$

The unit of the specific d-xylose uptake rate was given in mmol g_CDW_^−1^ h^−1^. All parameters were estimated from a single batch cultivation. To quantify the estimates’ uncertainties, we calculated the standard error of the parameter estimates, assuming that all parameter values are distributed normally. The standard errors of the derived parameters were automatically determined using the built-in error propagation formula of OriginPro (2023).

### 2.4 Molecular biology methods

Isolation of genomic DNA was performed using the NucleoSpin Microbial DNA kit, and plasmid isolation was performed with the NucleoSpin Plasmid (NoLid) kit from Macherey-Nagel (Düren, Germany) according to the manufacturer's instructions. Purification of PCR reactions and enzymatic restriction reactions was performed with the NucleoSpin Gel and PCR Clean-up kit from Macherey-Nagel. Restriction enzymes were obtained from New England Biolabs Inc. (Ipswich, Massachusetts, USA). PCR amplifications were performed with Q5 High-Fidelity DNA Polymerase 2x Master Mix from New England Biolabs according to the manufacturer's instructions. Annealing temperatures were calculated with the help of the NEB T_m_ calculator. Sequences of all PCR primers are listed in Table S4. *E. coli* DH5α was made chemically competent and transformed using the Inoue method (Green and Sambrook, 2020). Electrocompetent cells of *P. taiwanensis* VLB120 and *E. coli* DH5α λpir were prepared according to previously published methods (Choi et al., 2006; Sambrook and Russell, 2001). Electroporation was performed with electroporation cuvettes with a 2 mm gap in an electroporator (Equibio EasyjecT Prima, Kent, UK) at 2500 V. Colony PCRs were performed using Taq DNA polymerase 1.1x master mix RED (Ampliqon A/S, Odense M, Denmark) or Q5 High-Fidelity DNA Polymerase 2x Master Mix after cell lysis of single colonies in 30 μL alkaline polyethylene glycol as described before (Chomczynski and Rymaszewski, 2006). All new plasmid sequences were checked for correct assembly by sanger sequencing (Microsynth Seqlab GmbH, Göttingen, Germany).

#### 2.4.1 Plasmid and strain construction for lactonase gene expression

Plasmids for constitutive expression of the putative xylonolactonase genes were constructed by replacing the original expression cassette of pCom10lac by an artificial cassette adopted from plasmid pTN1_Syn35_Tra (Neves et al., 2020). To exchange the cassettes, the original expression cassette of pCom10lac was removed by PCR with the primers PPN103 and PPN104. The resulting linear PCR fragment was used as backbone in a subsequent Gibson cloning reaction (Gibson et al., 2009). The insert was generated by PCR with the primers PPN105 and PPN106. These primers harbored a 25 nt complementary region and were used to generate the 160 bp insert. After the assembly reaction chemically competent *E. coli* DH5α were transformed with 5 μL of Gibson reaction mixture. After incubation for 45 min in SOC medium, cells were plated on LB agar plates containing kanamycin overnight at 37 °C. Correct assembly was checked first by colony PCR with the primers SPPN039/SPPN040 and then by Sanger sequencing. Lactonase genes were cloned via Gibson cloning. Therefore, the previously generated empty vector plasmid pCom10Syn35T was linearized by PCR with the primers PPN107/PPN108. Genes coding for putative xylonolactonases were amplified from the genomic DNA of *P. taiwanensis* VLB120 with the primers PPN116/PPN117 (PVLB_05820) and PPN118/PPN119 (PVLB_12345). PCR products were purified and used in a Gibson assembly reaction. After transformation, correct plasmid assembly was first evaluated by colony PCR with the primers SPPN039/SPPN040 and then by Sanger sequencing.

#### 2.4.2 Plasmid and strain construction for transporter gene expression

Plasmids for expression of putative d-xylonate transporter genes were generated by Gibson cloning. Therefore, pCom10lac was linearized by enzymatic digest with *Nde*I at 37 °C for 3 h. After heat inactivation at 65 °C for 20 min, the fragment was purified by preparative agarose gel electrophoresis (1 % agarose, TAE buffer). Genes coding for putative d-xylonate transporters were amplified from genomic DNA of *Pseudomonas putida* KT2440 or *Pseudomonas taiwanensis* VLB120 with the primers PPN182/PPN183 (PP_3377/*kguT*), PPN184/PPN185 (PVLB_18545), PPN186/PPN187 (PVLB_13665/*gntP*). PCR products were purified and used in a Gibson assembly reaction. After transformation, correct assembly was first evaluated by colony PCR with the primers SPPN001/SPPN002 and then by Sanger sequencing.

#### 2.4.3 Gene deletions in *P. taiwanensis* VLB120

Knockout of transporter and regulatory genes was performed according to the protocol by (Martínez-García and de Lorenzo, 2011), which has been used successfully in *P. taiwanensis* VLB120 before (Volmer et al., 2014). Plasmids for recombination (pEMG_PVLB18545, pEMG_PVLB13665, pEMG_PVLB13655) were constructed by restriction cloning. Therefore, ∼500 bp of the flanking regions of the target knockout sequence were amplified and fused by overlap extension PCR using the corresponding primers (PVLB_18545 deletion: PPN170 – PPN173; PVLB_13665 deletion: PPN136 – PPN139; PVLB_13655 deletion: PPN132 – PPN135). The fused fragments were cloned into the pEMG backbone via the *Eco*RI and *Xba*I restriction sites. *E. coli* DH5α λpir was transformed with the ligated plasmid, plated on Km^50^ LB agar plates, and screened by colony PCR with the primers SPPN027/SPPN028. *P. taiwanensis* VLB120ΔC cells were transformed with the respective pEMG plasmids and plated on LB agar plates containing Sm^100^ and Km^50^. The successful integration was confirmed by colony PCR with respective primers (PVLB_18545 deletion: PPN170/PPN173; PVLB_13665 deletion: PPN136/PPN139; PVLB_13655 deletion: PPN132/PPN135) and based on the difference in PCR product size. Next, a positive clone was transformed with the plasmid pSW-2 to initiate I-SceI cleavage, and the second recombination event. After that, the cells were plated on LB agar plates containing Sm^100^ and Gm^25^. Replica plating was performed with Sm^100^ /Km^50^ and Sm^100^/Gm^25^ to sort out kanamycin-sensitive clones. Successful recombination was confirmed by colony PCR based on the difference in PCR product size and curing of pSW-2 was done by cultivation in LB medium without gentamicin. Loss of pSW-2 was verified via replica plating on Sm^100^ and Sm^100^/Gm^25^ agar plates. For multiple knockouts, the complete procedure was repeated accordingly.

### 2.5 Analytical methods

#### 2.5.1 Biomass quantification

For biomass quantification, a correlation of cell dry weight (CDW) to optical density at 450 nm (OD_450_) was determined. Cultures were grown according to the standard procedure described earlier (M9 medium with 20 g L^−1^ d-xylose). The cells were harvested by centrifugation at 4000×*g* for 20 min and resuspended in phosphate-buffered saline (PBS). Four dilutions with PBS were prepared, and the OD_450_ was determined five times each in a Libra S11 spectrophotometer (Biochrom Ltd., Cambridge, UK). Next, five samples, containing 9 mL each, were taken from each dilution and centrifuged as before. The supernatant was discarded, the cells were washed with 0.8 % NaCl solution and centrifuged again. Lastly, the cells were suspended in 1 mL ultrapure water and dried in weighed glass tubes at 70 °C until no change in weight was measurable. Values for optical density and dry weight were plotted, and the OD-biomass correlation factor was obtained by fitting a straight line with intercept 0 to the experimental data. An OD_450_ of 1 corresponded to 0.2049 g_CDW_ L^−1^ for *P. taiwanensis* VLB120ΔC and 0.2016 g_CDW_ L^−1^ for *P. taiwanensis* VLB120ΔCΔ*gntR* (Fig. S1).

#### 2.5.2 Quantification of d-xylose

The concentration of d-xylose was determined by an enzymatic assay kit from Megazyme Ltd. (Wicklow, Ireland). The assay was performed in transparent, flat-bottomed 96-well plates (Greiner Bio-One, Kremsmünster, Austria) according to the manufacturer's instruction in a FLUOstar-Omega microplate reader (BMG Labtech GmbH, Ortenberg, Germany) at 25 °C.

#### 2.5.3 Quantification of extracellular d-xylonate and d-xylonolactone

Quantification of d-xylonolactone and d-xylonate was performed with the hydroxamate assay (Lien, 1959). Originally used for the quantification of gluconolactone and gluconate, the assay is also applicable to quantify d-xylonolactone and d-xylonate (Dvořák et al., 2020; Dvořák and de Lorenzo, 2018; Köhler et al., 2015). After thawing of the frozen samples, the samples were kept at 4 °C. We found that long storage in an unfrozen state and multiple freeze-thaw cycles result in higher d-xylonate and lower d-xylonolactone concentrations consistent with the thermodynamic equilibrium. Hence, samples were measured directly after the first thawing. To determine the d-xylonolactone concentration, 30 μL H_2_O were mixed with 50 μL sample. Afterwards, 50 μL of the reaction mixtures were added to 100 μL hydroxylamine reagent (2 M hydroxylamine-hydrochloride in 2 M NaOH) in transparent, flat-bottomed 96-well plates. Next, 65 μl 3.2 M HCL and 50 μL FeCl_3_ (100 g in 0.1 M HCl) were added subsequently. The absorption at 550 nm was quantified within 10 min in a FLUOstar-Omega microplate reader. For determination of the cumulative d-xylonolactone and d-xylonate concentration, d-xylonate had to be converted to d-xylonolactone. Therefore, 30 μL 0.7 M HCl were mixed with 50 μL sample in the first step, and the reaction mixtures were heated to 99 °C for 15 min with constant shaking of 500 rpm in an Eppendorf Thermomixer C (Eppendorf, Hamburg, Germany). After cooling for 5 min in a water bath, reactions were treated as described above. Concentrations were quantified using a standard curve prepared with d-xylonolactone standards ranging from 0.1 g L^−1^ to 10 g L^−1^(Fig. S2). As the procedure with acidification and heating measures the cumulative concentration of d-xylonolactone and d-xylonate, the concentration of d-xylonate was calculated by subtraction of the obtained molar concentrations. As high concentrations of d-xylose interfered with the assay, an additional correlation of the d-xylose concentration with the absorption at 550 nm in the hydroxamate assay was generated (Fig. S3). Concentrations of d-xylose were measured with an enzymatic assay, and values for d-xylonolactone and d-xylonate were corrected for d-xylose values.

## 3 Results

### 3.1 Substrate dependent growth of *P. taiwanensis* VLB120 on d-xylose

**Fig. 2:**
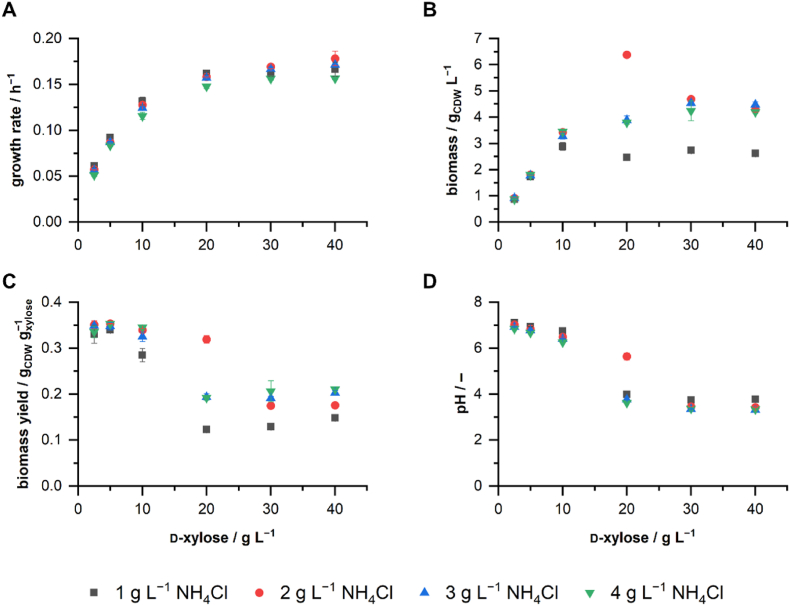
**Growth of *P. taiwanensis* VLB120ΔC at different****d****-xylose and ammonium chloride (NH**_**4**_**Cl) concentrations.** Growth rates during exponential growth **(A)** were determined from the backscatter signal of the cultures. Final biomass concentrations **(B)** and biomass yields **(C)** were calculated from OD_450_ measurements at the end of the cultivations. Final pH values **(D)** were measured at the end of the cultivations. Cultivations were performed in a BioLector I in modified M9 medium at 1 mL scale, 1200 rpm, 30 °C for 72 h. The mean values and error bars (standard deviations) were calculated from two biologically independent cultivations.

We cultivated *P. taiwanensis* VLB120ΔC on different concentrations of d-xylose to analyze the existing bottleneck of the Weimberg pathway in dependency of various nutritional and environmental conditions. The nitrogen source ammonium chloride was simultaneously supplied in four different concentrations to avoid growth limitation due to nitrogen availability under aerobic conditions. The Weimberg pathway activity was inferred from the growth rate, final biomass concentration, biomass yield, and the final pH of the cultures (Fig. 2).

The growth rate showed a high dependency on the initial concentration of d-xylose, and we observed Monod-shaped growth kinetics of *P. taiwanensis* VLB120 in dependency on the concentration of d-xylose (Fig. 2A). Nitrogen availability had a minor impact on the growth rate. As to be expected, the final biomass concentrations were also dependent on the initial concentrations of d-xylose (Fig. 2B). However, biomass formation in the standard M9 medium containing only 1 g L^−1^ NH_4_Cl appeared to be limited at around 3 g_CDW_ L^−^1. The highest biomass concentration was obtained with 20 g L^−1^ d-xylose and 2 g L^−1^ NH_4_Cl, which is a 260 % increase compared to the biomass obtained in M9 medium with 1 g L^−1^ NH_4_Cl. However, the growth was likely still nitrogen limited towards the end of cultivation since Weimberg pathway intermediates were still found in the medium (Fig. S4). Cultivation in M9 medium with 2 g L^−1^ NH_4_Cl or more and 30 or 40 g L^−1^ d-xylose resulted in less biomass formation than with 20 g L^−1^ d-xylose and 2 g L^−1^ NH_4_Cl, indicating another limiting, or even inhibiting factor. The biomass yields were influenced by the nitrogen source concentration but mainly by the concentration of d-xylose. (Fig. 2C). Low d-xylose concentrations resulted in the highest biomass yields of around 0.35 g_CDW_ g_xylose_^−1^. High concentrations of d-xylose resulted in decreased biomass yields and higher concentrations of residual d-xylose and pathway intermediates (Fig. S4). With 0.32 g_CDW_ g_xylose_^−1^, the cultivations with 20 g L^−1^ d-xylose and 2 g L^−1^ NH_4_Cl showed a higher biomass yield than the cultivations with 20 g L^−1^ d-xylose and other concentrations of NH_4_Cl. The pH decreased in all cultivations (Fig. 2D), which was likely caused by the extracellular accumulation of d-xylonate. This effect was more dominant at d-xylose concentrations above 10 g L^−1^ d-xylose. The pH of the cultivations with 20 g L^−1^ d-xylose and 2 g L^−1^ NH_4_Cl decreased to pH 5.6, comparatively less than in the cultivations with the same amount of d-xylose but with 3 or 4 g L^−1^ NH_4_Cl (pH 3.8 and 3.6, respectively). As the accepted pH range for *P. taiwanensis* VLB120 is between 4.5 and 10 (Köhler et al., 2015), the decreasing pH is likely be the cause of growth inhibition and final arrest. The results suggest a mechanistic relation between the biomass growth, the extracellular pH, and the Weimberg pathway metabolites d-xylonolactone and d-xylonate. As the metabolic activity of the Weimberg pathway resulted in considerable accumulation of intermediates and acidification of the medium, we further analyzed the pH as a major determinant of the Weimberg pathway activity.

### 3.2 Influences of static pH on growth and Weimberg pathway

**Fig. 3:**
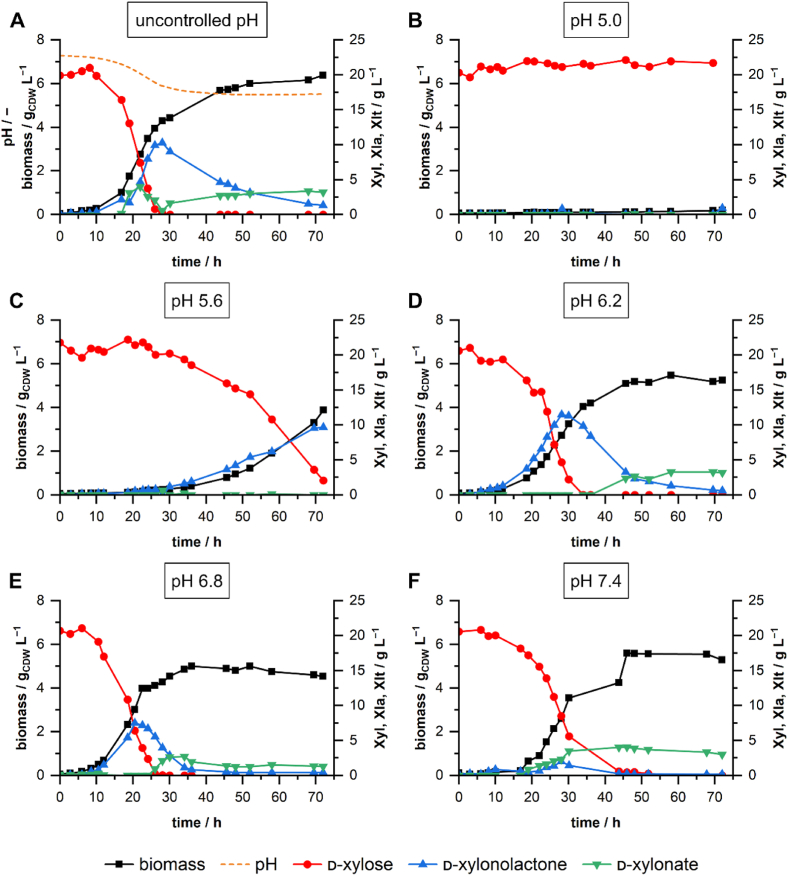
**Bioreactor cultivations of *P. taiwanensis* VLB120ΔC with and without** pH **control.** Uncontrolled cultivation with initial pH of 7.4 **(A)** and pH-controlled cultivations at pH 5.0 **(B)**, pH 5.6 **(C)**, pH 6.2 **(D)**, pH 6.8 **(E)**, pH 7.4 **(F)** were performed in 200 mL M9 medium (20 g L^−1^d-xylose and 2 g L^−1^ NH_4_Cl) in DASbox bioreactors. The batch bioreactors were operated at 30 °C, 1000 rpm, and aeration of 0.25 vvm. The pH was controlled with 1 M NaOH and 1 M H_3_PO_4_.

As the growth on increasing concentrations of d-xylose was accompanied by a strong drop in pH, we assessed the growth behavior under different static pH conditions with 20 g L^−1^ d-xylose and 2 g L^−1^ NH_4_Cl (Fig. 3). Therefore, we cultivated *P. taiwanensis* VLB120 in stirred-tank bioreactors (STRs) without pH control and at 5 different static pH conditions (pH 5.0–7.4). When cultivated under uncontrolled conditions, the pH dropped strongly until d-xylose was depleted. During exponential growth, d-xylonolactone transiently accumulated in the medium up to a concentration of around 10 g L^−1^ between 25 and 30 h. After that, d-xylonolactone was gradually taken up again. Extracellular d-xylonate also accumulated but to a much lower extent with a local maximum concentration between 20 and 25 h of cultivation. After the depletion of d-xylose, the d-xylonate concentration in the medium slowly increased until the end of cultivation. Growth behavior and accumulation of Weimberg pathway intermediates at static pH conditions differed considerably from the uncontrolled pH profile. While at pH 5, only very slow growth occurred, *P. taiwanensis* VLB120 was able to grow considerably on d-xylose at all other tested pH values between 5.6 and 7.4. The highest growth rate of 0.206 h^−1^ was observed at pH 6.8, suggesting an optimal pH with regard to growth rate in the range between 6.2 and 7.4. Moreover, higher growth rates correlated with earlier depletion of d-xylose. Except at pH 5 all cultivations showed extracellular accumulation of d-xylonolactone and d-xylonate. Accumulation of d-xylonolactone occurred during exponential growth (Fig. 3 CDE). Accumulation of d-xylonate started after depletion of d-xylose (Fig. 3 DE). Static pH values below 7.4 enforced accumulation of d-xylonolactone, with a maximum concentration of ∼11.5 g L^−1^ during growth at pH 6.2. A pH value of 6.8 led to reduced accumulation, and at pH 7.4, the maximum d-xylonolactone concentration was only approximately 2 g L^−1^. Our findings suggest not only the accumulation of d-xylonate as a bottleneck during the growth of *P. taiwanensis* VLB120 on d-xylose but especially its prior metabolite d-xylonolactone. This might be due to insufficient autohydrolysis and lacking or low activity of a yet unknown xylonolactonase.

### 3.3 Tackling the bottleneck of d-xylonolactone hydrolysis

**Fig. 4:**
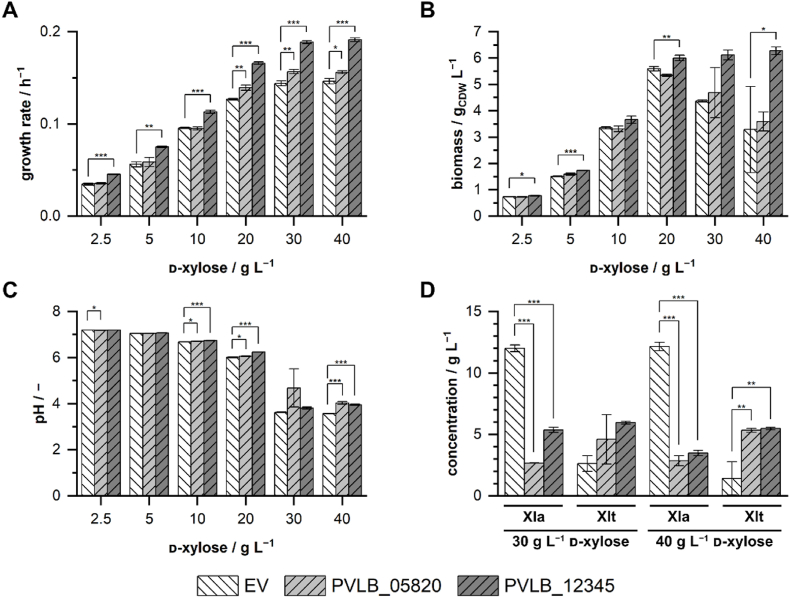
**Cultivations of *P. taiwanensis* VLB120ΔC with plasmid-based overexpression of putative lactonase genes.** Growth rates during exponential growth **(A)** were determined from the backscatter signal. Final biomass concentrations **(B)** were calculated from OD_450_ measurements at the end of the cultivations. Final pH values **(C)** and final metabolite concentrations of d-xylonolactone (Xla) and d-xylonate (Xlt) **(D)** were measured at the end of the cultivations. Cultivations of the empty vector control (EV) and the strains overexpressing PVLB_05820 and PVLB_12345 were performed in a BioLector I in M9 medium (varying concentrations of d-xylose, 2 g L^−1^ NH_4_Cl) at 1 mL scale, 1200 rpm, 30 °C for 72 h. The corresponding growth curves are depicted in Fig. S5. The mean values and error bars (standard deviations) were calculated from at least two biologically independent cultivations. One-way ANOVA tests including post hoc analyses (Bonferroni) were performed for the mean values. Asterisks denote statistically significant difference between two mean values: p ≤ 0.05 (*), p ≤ 0.01 (**), p ≤ 0.001 (***). The level of significance between the engineered strains and the empty vector control is depicted. The detailed statistical report of the one-way ANOVA analysis is shown in the supplementary material (Table S6 to Table S9).

To assess whether insufficient xylonolactonase activity generates a bottleneck in the Weimberg pathway, we overexpressed two endogenous genes coding for putative lactonases from *P. taiwanensis* VLB120. The annotated gluconolactonase encoded by PVLB_05820 represents the ortholog of the gluconolactonase encoded by PP_1170, which is hypothesized to catalyze the reaction in *Pseudomonas putida* strains (Bator et al., 2019; Dvořák et al., 2020; Meijnen et al., 2009). The putative lactonase gene PVLB_12345 was identified by Protein BLAST analysis of XylC from *Caulobacter crescentus* and the *P. taiwanensis* VLB120 genome (28.18 % identity). The strains with plasmid-based overexpression of the lactonases, and an empty vector control strain were cultivated in a BioLector in M9 medium containing different d-xylose concentrations (Fig. 4).

As observed before in chapter 3.1, the growth rate increased with increasing concentrations of d-xylose (Fig. 4A). Generally, the growth rates of the empty vector control strain were lower compared to the non-plasmid baring strain (Fig. 2). In cultivations with concentrations of d-xylose of 20 g L^−1^ and above, *P. taiwanensis* VLB120 with constitutive overexpression of PVLB_05820 showed a slightly higher growth rate compared to the EV (empty vector) control (7–10 %). Overexpression of PVLB_12345 led to increased growth rates at all tested initial d-xylose concentrations. The growth rate with pCom10_Syn35T_PVLB12345 increased by 18 % for 10 g L^−1^ d-xylose and by 30 % for all other tested concentrations. The generated biomass of the three strains at the end of the cultivations was very similar up to an initial d-xylose concentration of 20 g L^−1^ (Fig. 4B). At 30 and 40 g L^−1^ d-xylose, strains harboring pCom10_Syn35T_PVLB12345 reached a higher biomass concentration than the two other strains. The highest biomass concentration was found at a d-xylose concentration of 40 g L^−1^, where *P. taiwanensis* VLB120ΔC pCom10Syn35T_PVLB12345 reached 6.3 g_CDW_ L^−1^, which was almost double the biomass concentration of the strain with pCom10_Syn35T_PVLB05820 and the empty vector control. Moreover, as observed before, the extracellular pH dropped depending on the initial concentration of d-xylose (Fig. 4C). At 30 and 40 g L^−1^, the pH of all cultivations dropped to around 4. The final concentrations of the Weimberg pathway intermediates showed that overexpression of the two lactonases significantly lowered the extracellular concentration of d-xylonolactone compared to the empty vector control strain (Fig. 4D). In accordance with that, increased concentrations of d-xylonate were found in the medium. The results support our hypothesis of insufficient lactonase activity of *P. taiwanensis* VLB120 as a bottleneck for growth via the Weimberg pathway. Moreover, the increase of extracellular d-xylonate indicates another potential bottleneck for further optimization. Hence, we next examined the uptake of d-xylonate in *P. taiwanensis* VLB120.

### 3.4 Tackling the bottleneck of d-xylonate uptake

**Fig. 5:**
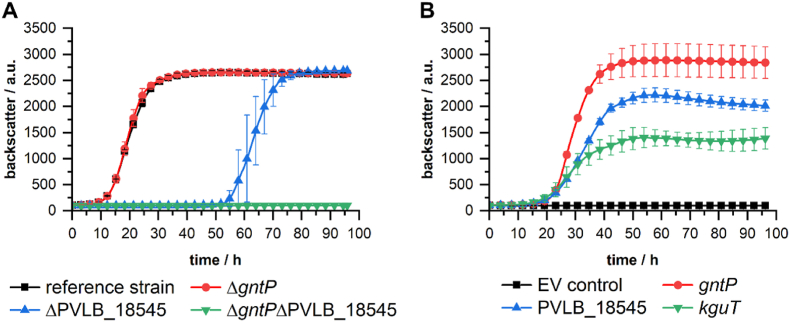
**Complementation assay of genes potentially relevant for****d****-xylonate transport in *P. taiwanensis* VLB120.** Growth experiments with *P. taiwanensis* VLB120ΔC (reference strain) and the knockout strains *P. taiwanensis* VLB120ΔCΔ*gntP*, *P. taiwanensis* VLB120ΔCΔPVLB_18545 and *P. taiwanensis* VLB120ΔCΔ*gntP*ΔPVLB_18545 **(A).** The double-knockout strain *P. taiwanensis* VLB120ΔCΔ*gntP*ΔPVLB_18545 was complemented with plasmid-based overexpression of the transporter genes *gntP*, PVLB_18545, and *kguT***(B)**. All cultivations were performed in M9 medium (20 g L^−1^d-xylose and 2 g L^−1^ NH_4_Cl) at 25 mL scale, 30 °C and 200 rpm. The mean values and error bars (standard deviations) were calculated from two biologically independent cultivations.

Although d-xylonate accumulation was previously reported for *P. taiwanensis* VLB120 and other pseudomonads, to this date, no xylonate transporter has been verified for *P. taiwanensis* VLB120. The gluconate transporter GntP (PP_3417) was hypothesized in earlier studies as a transporter in *Pseudomonas putida* strains (Bator et al., 2019; Meijnen et al., 2009). Recently, the 2-ketogluconate transporter KguT (PP_3377) was proven to facilitate d-xylonate transport in *P. putida* KT2440 (Lim et al., 2021). However, *P. taiwanensis* VLB120 does not harbor an ortholog of *kguT* and the other genes from the ketogluconate loop, *kguD*, *gnuK,* and *kguK* (Köhler et al., 2013). Protein BLAST analysis of the amino acid sequence of KguT and the genome of *P. taiwanensis* VLB120 revealed 4 homologs of the major facilitator superfamily (MFS) (Table S5). Of those four homologs, the MFS transporter encoding gene PVLB_18545 caught our attention as no ortholog is present in *P. putida* KT2440. Moreover, the gene is located in close proximity to other genes from the Weimberg pathway on the genome of *P. taiwanensis* VLB120 (Fig. S7). To identify the d-xylonate transporter in *P. taiwanensis* VLB120, we generated knockout strains of the gluconate transporter GntP as well as PVLB_18545 and tested them for their ability to grow on d-xylose (Fig. 5).

Knockout of the gluconate transporter GntP did not change the growth behavior compared to the reference strain (Fig. 5A). This indicates that other transporters are still sufficient to transport d-xylonate and to sustain growth. Additional knockout of the gene PVLB_18545 in *P. taiwanensis* VLB120ΔCΔ*gntP* led to the complete absence of growth during almost 100 h of cultivation. The single knockout strain *P. taiwanensis* VLB120ΔCΔPVLB_18545 was able to grow after an extensive lag phase of around 50 h. To verify the involvement of *gntP* and PVLB_18545 in d-xylonate transport, the double-knockout strain *P. taiwanensis* VLB120ΔCΔ*gntP*ΔPVLB_18545 was complemented with the genes coding for the transporters KguT, GntP, and PVLB_18545. Transporter activity was followed by cultivating the strains on d-xylose (Fig. 5B). While the empty vector control strain could not grow on d-xylose, expression of *kguT* restored the ability to grow on d-xylose. The two endogenous transporters also restored growth, confirming our results with the knockout strains. GntP and the MFS transporter encoded by PVLB_18545 exhibit the ability to transport d-xylonate. Our data suggest that both transporters, GntP and PVLB_18545, transport d-xylonate; however, the characteristics of the transporters are apparently quite different.

**Fig. 6:**
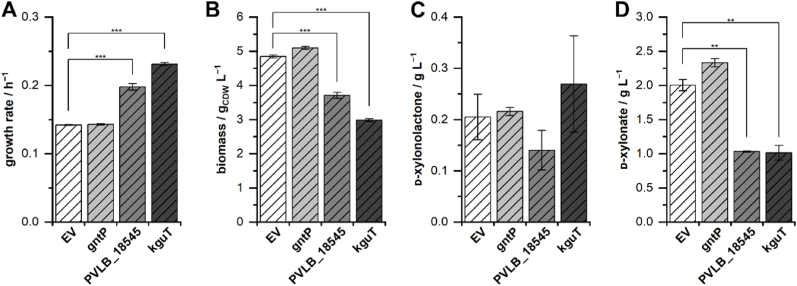
Transporter gene overexpression in *P. taiwanensis* VLB120ΔC. Panels depict the initial growth rates of the exponential growth phases (A), final biomass concentrations (B), final d-xylonolactone concentrations (C) and final d-xylonate concentrations (D). Cultivations of the empty vector control (EV) and the strains overexpressing *gntP*, PVLB_18545, and *kguT* were performed in a BioLector I in M9 medium (20 g L^−1^d-xylose and 2 g L^−1^ NH_4_Cl) at 1 mL scale, 1200 rpm, 30 °C for 96 h. The growth rate was calculated from the backscatter signal. For the final biomass concentration, the OD_450_ was measured. The mean values and error bars (standard deviations) were calculated from two biologically independent cultivations. One-way ANOVA tests including post hoc analyses (Bonferroni) were performed for the mean values. Asterisks denote statistically significant difference between two mean values: p ≤ 0.05 (*), p ≤ 0.01 (**), p ≤ 0.001 (***). The level of significance between the engineered strains and the empty vector control is depicted. The detailed statistical report of the ANOVA analysis is shown in the supplementary material (Table S10).

Having identified new d-xylonate transporters, we tested whether overexpression of the respective transporter genes in the parent strain *P. taiwanensis* VLB120ΔC can further improve the overall growth performance. Therefore, we overexpressed *gntP*, PVLB_18545 and *kguT* in *P. taiwanensis* VLB120ΔC in growth experiments on d-xylose in the BioLector. Overexpression of *gntP* showed only little deviation from the empty vector control concerning the growth rate (Fig. 6). In contrast to that, overexpression of PVLB_18545 resulted in the occurrence of two growth phases, the first exhibiting an increased and the second a decreased growth rate (the higher initial growth rate is depicted in Fig. 6). Overexpression of *kguT* led to higher initial growth rates, which then decreased over time. The final biomass concentration of the empty vector control and the strain expressing *gntP* was ∼5 g_CDW_ L^−1^, while the other two strains only reached a concentration of ∼3.7 g_CDW_ L^−1^ (PVLB_18545) and ∼3 g_CDW_ L^−1^ (*kguT*). The pH reached a final value of around 6 in all cultivations (not shown). The cultivations of the four strains differed in the concentrations of the remaining Weimberg pathway metabolites at the end of the cultivations. The concentrations of d-xylonolactone were similar at around 0.2 g L^−1^, and the concentrations of d-xylonate were decreased by around 50 % with overexpression of PVLB_18545 and *kguT* in contrast to the empty vector control, demonstrating an increased uptake of d-xylonate facilitated by the transporters.

Although d-xylonate uptake was increased with overexpression of PVLB_18545 and *kguT*, the total biomass concentrations decreased, which might be caused by stress due to overexpression of the transporter genes. Moreover, we observed biphasic growth with strongly different growth rates when PVLB_184545 was overexpressed (Fig. S6). Therefore, we sought other ways to improve d-xylonate transport. To our knowledge, nothing is known about the regulation of the expression of PVLB_18545. However, the expression of the gluconate transporter gene *gntP* was studied in *Pseudomonas aeruginosa* PAO1. In this strain, *gntP* is regulated by the repressor protein GntR (Daddaoua et al., 2017). GntR binds to its own and the *gntP* promoter region to repress gene expression. Moreover, GntR works in an effector-mediated de-repression mechanism caused by glucose, gluconate, and 6-phosphogluconate. Orthologs of the genes are also present in *P. taiwanensis* VLB120 (Fig. S8). Hence, a knockout of *gntR* might lead to an increased expression of GntP and we applied transcription factor engineering for improving d-xylonate uptake by knocking out *gntR* (PVLB_13655). In a next step, we therefore analyzed the effect of the *gntR* knockout and overexpression of PVLB_12345 in stirred-tank bioreactors.

### 3.5 Evaluation of single and combinatorial optimization strategies in stirred-tank bioreactors

**Fig. 7:**
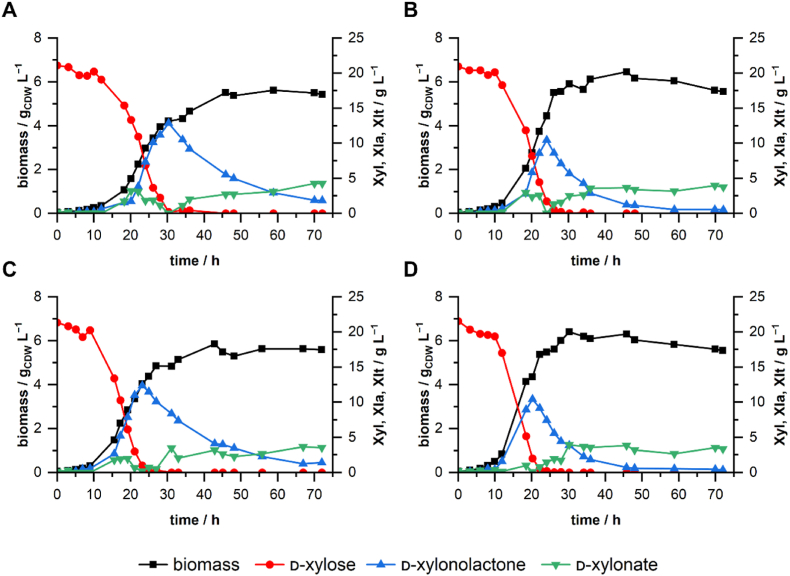
**Bioreactor cultivations of single and combinatorial optimization approaches.***P. taiwanensis* VLB120ΔC containing pCom10Syn35T (empty vector control) **(A)**, *P. taiwanensis* VLB120ΔC pCom10Syn35T_PVLB12345 **(B)**, *P. taiwanensis* VLB120ΔCΔ*gntR* pCom10Syn35T **(C)** and *P. taiwanensis* VLB120ΔCΔ*gntR* pCom10Syn35T_PVLB_12345 **(D)** were cultivated in 200 mL M9 medium (20 g L^−1^d-xylose and 2 g L^−1^ NH_4_Cl). Cultivations were performed in the DASbox over 75 h at 30 °C at 1000 rpm and an aeration of 3 L h^−1^.

In comparison to the empty vector control strain, we cultivated *P. taiwanensis* VLB120ΔC with plasmid-based overexpression of the lactonase gene PVLB_12345, *P. taiwanensis* VLB120ΔC with the deregulated gluconate transporter gene *gntP* and a strain with a combination of the *gntP* deregulation and PVLB_12345 overexpression. The results demonstrate the improvements of the single engineering strategies and the combinatorial approach (Fig. 7). During exponential growth, d-xylose was taken up, and d-xylonolactone accumulated in the medium. For the control strain *P. taiwanensis* VLB120ΔC pCom10Syn35T, d-xylose was depleted after ∼30 h and in the cultivations of the engineered strains *P. taiwanensis* VLB120ΔC pCom10Syn35_PVLB12345, and *P. taiwanensis* VLB120ΔCΔ*gntR* pCom10Syn35, d-xylose was depleted after 28 h and 25 h respectively. *P. taiwanensis* VLB120ΔCΔ*gntR* pCom10Syn35_PVLB12345 consumed d-xylose even faster, within 22–24 h. This correlates to the specific growth rates and specific xylose uptake rates determined for the strains (Table 1). All engineered strains showed increased growth rates in comparison to the empty vector strain. The maximum d-xylonate concentrations were very similar in the cultivations, ranging between 3.4 and 4.3 g L^−1^. d-Xylonolactone accumulation was strongly influenced by the overexpression of the lactonase gene PVLB_12345. Overexpression of PVLB_12345 reduced the transient accumulation of d-xylonolactone in the medium and increased the strain's growth rate. While in the control strain cultivation, d-xylonolactone reached its maximum concentration after 30 h with 13 g L^−1^, overexpression of PVLB_12345 led to a peak after 24 h of 10.5 g L^−1^. The strain *P. taiwanensis* VLB120ΔCΔ*gntR* pCom10Syn35 showed a maximum concentration of 12.4 g L^−1^ after 23 h, and *P. taiwanensis* VLB120ΔCΔ*gntR* pCom10Syn35_PVLB12345 had its maximum concentration of d-xylonolactone already after 20 h with 10.4 g L^−1^. The knockout of *gntR* had positive influence on the growth rate and led to a slightly earlier accumulation of d-xylonolactone. The engineered strain *P. taiwanensis* VLB120ΔCΔ*gntR* pCom10Syn35_PVLB12345 showed an improved growth rate and biomass yield of 50 % and 24 %, respectively, in contrast to the empty vector control strain.

**Table 1:** **Growth rates and biomass yields**. Strains were cultivated in 200 mL M9 medium (20 g L^−1^d-xylose and 2 g L^−1^ NH_4_Cl). Cultivations were performed in DASbox bioreactors over 75 h at 30 °C at 1000 rpm and an aeration of 3 L h^−1^. Parameters μ (growth rate), Y_X/S_ (specific biomass yield) and q_S_ (specific substrate uptake rate) including standard errors of the parameter estimates were determined in a nonlinear regression analysis using the time course data of the exponential growth phases during the single batch bioreactor cultivations.

Strain	μ/h^−1^	Y_X/S_/g_CDW_ g_d-xylose_^−1^ (exponential phase)	q_S_/mmol g_CDW_^−1^ h^−1^
*P. taiwanensis* VLB120ΔC EV	0.16 ± 0.01	0.21 ± 0.02	5.15 ± 0.40
*P. taiwanensis* VLB120ΔC PVLB_12345	0.21 ± 0.02	0.22 ± 0.02	6.32 ± 0.64
*P. taiwanensis* VLB120ΔCΔ*gntR* EV	0.18 ± 0.02	0.18 ± 0.03	6.53 ± 0.96
*P. taiwanensis* VLB120ΔCΔ*gntR* PVLB_12345	0.24 ± 0.02	0.26 ± 0.02	6.02 ± 0.55

## 4 Discussion

This study focused on the identification of bottlenecks and associated engineering targets of *P. taiwanensis* VLB120 during growth on d-xylose via the Weimberg pathway. The accumulation of d-xylonolactone and d-xylonate in the cultivation medium suggested metabolic bottlenecks in the periplasmic reactions, i.e., d-xylonolactone hydrolysis and d-xylonate uptake. Accumulation of d-xylonolactone has been described for *P. fragi* growing on d-xylose by Weimberg (1961) and later by Buchert (Buchert and Viikari, 1988) but not yet for *P. taiwanensis* VLB120. As in our experiments, d-xylonolactone accumulated transiently in these studies. The reasons for the accumulation of d-xylonolactone are yet unknown. However, due to its uncharged nature, d-xylonolactone is more likely to diffuse out of the periplasm than the dissociated acid d-xylonate. Low activity of the xylonolactonase or insufficient expression of the corresponding gene might be reasonable explanations. Although the reaction of d-xylonolactone to d-xylonate can occur non-enzymatically, the reaction can be limiting at higher Weimberg pathway fluxes (Shen et al., 2020). In our study, overexpression of two genes coding for putative lactonases PVLB_05820 and PVLB_12345 led to a reduction in the final d-xylonolactone concentration and increased growth rates, which indicates optimized metabolic flux. These lactonases are likely active for d-gluconolactone and d-xylonolactone since the enzymes from the oxidative carbon metabolism of *Pseudomonas* accept different sugars and their corresponding metabolites as substrates. Moreover, the xylonolactonase from *C. crescentus* XylC is active for d-gluconolactone and d-xylonolactone (Pääkkönen et al., 2021).

The equilibrium reaction between d-xylonolactone and d-xylonate is dependent on the pH (Hummel et al., 2010). Thus, the change of the pH during cultivation will shift the equilibrium between the lactone and the acid. A pH value above 7 strongly favors the dissociated acid form and thus restricts lactonization, while a lower pH value leads to increased formation of the lactone species (Hummel et al., 2010). Direct measurements of the periplasmic pH in *E. coli* have shown that the periplasmic pH is highly dependent on the extracellular pH and that the outer membrane does not pose a significant barrier to proton movement (Wilks and Slonczewski, 2007). Hence, it is likely that the extracellular pH strongly influences the pH in the periplasm of *P. taiwanensis* VLB120. In our study, bioreactor cultivations with static pH conditions showed that a pH value of 7.4 reduced the accumulation of d-xylonolactone in the medium remarkably in contrast to lower pH values. This might be attributed to the equilibrium, which is strongly on the side of d-xylonate at pH 7.4. Cultivation without pH control led to acidification of the medium, which changes the equilibrium concentrations over time. Control of the cultivation pH thus might present an option to control Weimberg pathway flux in bioprocesses with *P. taiwanensis* VLB120. Acidification of the culture medium is generally a known phenomenon for *Pseudomonas*. In oxidative glucose metabolism, efflux of gluconic acid leads to acidification of the environment and is hypothesized to have evolved due to the environmental conditions of the natural rhizospheric habitats to enable solubilization of inorganic phosphate under phosphate-limiting conditions (Blank, 2012; Buch et al., 2008; de Werra et al., 2009). Moreover, the conversion withdraws the simple sugar from the environment and creates unfavorable conditions for competing microorganisms. It is reasonable that similar effects apply to the Weimberg pathway as oxidative d-xylose pathway. Furthermore, having the first two reactions take place in the periplasmic space might be advantageous to protect the cytoplasm from fatal changes of the intracellular pH, which occurred, for example, in engineered *S. cerevisiae* with expression of xylose dehydrogenase and xylonolactonase from *C. crescentus* (Nygård et al., 2014).

We successfully identified two transporters of *P. taiwanensis* VLB120 with the ability to transport d-xylonate. Our results suggest a major role of the MFS transporter protein encoded by PVLB_18545 in d-xylonate uptake, as knockout of *gntP* had no influence on the growth phenotype. However, GntP alone can fully compensate for the loss of the gene PVLB_18545, indicating a complex cellular regulation, different affinities of the transporters for d-xylonate, or different expression levels of the respective genes. The prolonged lag phase of the PVLB_18545 knockout strain indicated a slow adaptation of the knockout strain to the culture conditions due to insufficient transporter level after inoculation. This is further supported by the fact that *gntP* is not induced by d-xylose but rather by d-glucose (Köhler et al., 2015). Although we identified GntP as a d-xylonate transporter, it had not been identified by Lim et al. as a transporter in *P. putida* KT2440. In this strain, KguT is the main facilitator of d-xylonate transport (Lim et al., 2021). However, it is likely that GntP of *P. putida* KT2440 can also transport d-xylonate, as the orthologous enzymes of the two strains share 95.3 % sequence identity. This is supported by another study, where *kguT* was knocked out in engineered *P. putida* KT2440 growing via the Weimberg pathway and the strain could still grow after a very long lag phase (Bator, 2021). Plasmid-based overexpression of the transporter genes did not increase overall strain performance. Although overexpression of *kguT* and PVLB_18545 led to increased growth rates, they were not sustained for long, and the biomass concentrations were decreased at the end of the cultivations. Notably, less d-xylonate was found at the end of the experiment, verifying the role as d-xylonate transporters. The higher level of transporter proteins might likely lead to destabilization of the membrane and similar side effects on growth. Moreover, general plasmid maintenance and metabolic burden due to the expression level might explain the lower performance. Cultivation in stirred-tank bioreactors showed an improved growth rate with a *gntR* knockout strain. Thus, deregulation of GntP is a preferred option to plasmid-based overexpression of the transporter genes. However, the different roles of the two transporters remain to be elucidated. The novel d-xylonate transporters require further investigations to reveal mechanistic properties of the transport in *Pseudomonas* strains and to develop, for example, protein engineering strategies for further strain optimization.

We observed that the growth is highly dependent on the concentration of d-xylose, and high concentrations (above 20 g L^−1^ d-xylose) are needed for maximum growth rates. This could be a result of the affinity of the periplasmic glucose dehydrogenase Gcd (PVLB_05240), which performs the conversion of d-xylose. The affinity of Gcd is higher for d-glucose than for d-xylose (Köhler et al., 2015). Hardy et al. determined an apparent K_M_ of Gcd from *P. putida* NCTC 10936 of 1–2 mM for glucose and 17–20 mM for d-xylose (Hardy et al., 1993). Another study showed that the activity for d-xylose is only 13–16 % of the activity for d-glucose of the periplasmic glucose dehydrogenase from a *Pseudomonas fluorescens*-type strain (Matsushita et al., 1980). Hence, the Weimberg pathway with native Gcd limits utilization of glucose and xylose mixtures, such as in lignocellulosic hydrolysates. If co-utilization of the sugars is desired, engineering of Gcd or introduction of a dedicated periplasmic xylose dehydrogenase would be necessary. Alternatively, deletion of Gcd and introduction of a xylose transporter gene and genes coding for intracellular xylose dehydrogenase and xylonolactonase (e.g., from *C. crescentus*) might be an option.

In our study, the pH of the medium influenced the growth *P. taiwanensis* VLB120. Growth rates were increased at pH 6.8 compared to pH 7.4 with similar starting concentrations of d-xylose, which might be a result of the pH optimum of Gcd. In cultivations without pH control, the decreasing pH caused by the accumulation of d-xylonate might have first led to more optimal reaction conditions for Gcd, and thus an accelerated conversion of d-xylose. The pH optimum of the enzyme is not known, but an optimal pH for growth was found to be around pH 6.5 for *P. fragi* (Buchert et al., 1986). For this strain a pH below 5 resulted in arrest of the growth, the d-xylose consumption, and the formation of d-xylonate. In our experiments, *P. taiwanenesis* VLB120 did not grow at a pH of 5 and no accumulation of d-xylonolactone or d-xylonate was detectable during 72 h of cultivation, which is in line with the results obtained for *P. fragi*.

In this study, we set out to identify bottlenecks of the Weimberg pathway and find ways to encounter them. Our metabolic engineering efforts led to increased growth rates and biomass yields, but the pathway is still far from being optimized. The Weimberg pathway requires further improvement for the establishment of a real bioprocess scenario because the pathway capacity influences the space-time yields and production yields, which are critical metrics in bioprocess development. Firstly, the utilization of *P. taiwanensis* VLB120ΔC, harboring a streptomycin resistance with the addition of plasmid-based overexpression with our genes of interest, is likely to pose a decent amount of metabolic burden already. Therefore, fine-tuning the expression of the Weimberg pathway genes on the genome will certainly be beneficial for further strain engineering. Utilization of a streamlined, genome-reduced *P. taiwanensis* VLB120 mutant strain might also lead to improved performance on d-xylose (Wynands et al., 2019). Secondly, the drain of Weimberg pathway intermediates for product formation will likely influence the pathway fluxes. Future research should therefore include the synthesis of a product molecule in order to explore the capacity of the Weimberg for product generation in addition to the microbial biomass.

## 5 Conclusion

In this study, we identified two bottlenecks of *P. taiwanensis* VLB120 during growth on d-xylose and employed rational metabolic engineering strategies for the optimization. Xylonolactonase activity, d-xylonate import, and the extracellular pH represent crucial factors in the pathway activity. Especially overexpression of the endogenous xylonolactonase gene PVLB_12345 improved the growth on d-xylose and reduced the accumulation of d-xylonolactone. Moreover, we identified two novel transporters with the ability to transport d-xylonate, the protein encoded by PVLB_18545 and the gluconate transporter GntP. Our results shed light on the yet fragmented knowledge of the Weimberg pathway in *Pseudomonas* strains and set the stage for future engineering of *P. taiwanensis* VLB120 or other pseudomonads for improved growth on the renewable carbon source d-xylose.

## Funding

This research was supported by the CLIB-Competence Center Biotechnology (CKB), funded by the 10.13039/501100008530European Regional Development Fund (10.13039/501100008530EFRE) and the North-Rhine Westphalian 10.13039/501100004725Ministry of Economic Affairs, Innovation, Digitalization and Energy (10.13039/501100016377MWIDE) [Grant number: EFRE-0300098].

## CRediT authorship contribution statement

**Philipp Nerke:** Writing – review & editing, Writing – original draft, Visualization, Validation, Methodology, Investigation, Formal analysis, Data curation, Conceptualization. **Jonas Korb:** Writing – review & editing, Visualization, Validation, Investigation, Formal analysis. **Frederick Haala:** Validation, Investigation. **Georg Hubmann:** Writing – review & editing, Supervision, Conceptualization. **Stephan Lütz:** Writing – review & editing, Supervision, Resources, Project administration, Funding acquisition, Data curation, Conceptualization.

## Declaration of competing interest

The authors declare that they have no known competing financial interests or personal relationships that could have appeared to influence the work reported in this paper.

## Data Availability

Data will be made available on request.
