# Supplementary material for: Metabolic bottlenecks of Pseudomonas taiwanensis VLB120 during growth on d-xylose via the Weimberg pathway
Source: Metab Eng Commun. 2024 Jun 6;18:e00241. doi: 10.1016/j.mec.2024.e00241 (PMC11252243; doi:10.1016/j.mec.2024.e00241)
Supplement: Multimedia component 1 [file mmc1.docx]

**Supplementary Information**

**Metabolic bottlenecks of *Pseudomonas taiwanensis* VLB120 during growth on d-xylose via the Weimberg pathway**

Philipp Nerke, Jonas Korb, Frederick Haala, Georg Hubmann, and Stephan Lütz*

*Chair for Bioprocess Engineering, Department of Biochemical and Chemical Engineering, TU Dortmund University, Emil-Figge-Straße 66, 44227 Dortmund, Germany*

*Corresponding author. E-mail: stephan.luetz@tu-dortmund.de

Table of contents

[Table S1: Growth media and solutions. 3](#_Toc167746433)

[Table S2: Strains. 3](#_Toc167746434)

[Table S3: Plasmids. 4](#_Toc167746435)

[Table S4: Primers. 5](#_Toc167746436)

[Figure S1: Correlation of optical density at 450 nm (OD_450_) and biomass concentration cell dry weight (CDW). 7](#_Toc167746437)

[Figure S2: Correlation between absorption at 550 nm and d-xylonolactone concentration (hydroxamate assay). 7](#_Toc167746438)

[Figure S3: Correlation between absorption at 550 nm and d-xylose concentration (hydroxamate assay). 7](#_Toc167746439)

[Figure S4: Final extracellular substrate and metabolite concentrations of BioLector cultivations with different concentrations of d-xylose and ammonium chloride (NH_4_Cl). 8](#_Toc167746440)

[Figure S5: BioLector growth curves of cultivations with plasmid-based overexpression of putative lactonase genes. 9](#_Toc167746441)

[Table S5: Protein BLAST search for the amino acid sequence of KguT in the genome of *P. taiwanensis* VLB120. 10](#_Toc167746442)

[Figure S6: BioLector growth curves of cultivations with plasmid-based overexpression of xylonate transporter genes. 10](#_Toc167746443)

[Figure S8: Deregulation of GntP in *P. taiwanensis* VLB120ΔC. 11](#_Toc167746444)

[Table S6: Post hoc analysis data for the growth rates of Figure 4. 12](#_Toc167746445)

[Table S7: Post hoc analysis data for the biomass concentrations of Figure 4. 13](#_Toc167746446)

[Table S8: Post hoc analysis data for the pH values of Figure 4. 14](#_Toc167746447)

[Table S9: Post hoc analysis data for the concentrations of d-xylonolactone and d-xylonate of Figure 4. 15](#_Toc167746448)

[Table S10: Post hoc analysis data for the mean values presented in Figure 6. 16](#_Toc167746449)

[Table S11: Biomass and d-xylose concentrations from stirred-tank bioreactor experiments presented in Figure 7. 17](#_Toc167746450)

[Cited literature 18](#_Toc167746451)

## Table S1: Growth media and solutions.

| Media and solutions | Components | Reference |
| --- | --- | --- |
| LB-medium  (plates) | 10 g L^−1^tryptone, 5 g L^−1^ yeast extract, 10 g L^−1^ NaCl, (15 g L^−1^ agar agar) | (Bertani, 1951) |
| M9 medium | 8.5 g L^−1^ Na_2_HPO_4_∙ H_2_O, 3 g L^−1^ KH_2_PO_4_, 0.5 g L^−1^NaCl, 1 – 4 g L^−1^ NH_4_Cl, 2 mL L^−1^ 1 MgSO_4_, 1 mL L^−1^ US^Fe^‑trace element solution, pH 7.4, 2.5 – 40 g L^−1^ d‑xylose | (Sambrook and Russell, 2001) |
| US^Fe^‑trace element solution | 82.81 mL 37 % fuming HCL, 8.87 g L^−1^ FeSO_4_∙ 7 H_2_O, 4.12 g L^−1^ CaCl_2_∙ 2 H2O, 1.5 g L^−1^ MnCl_2_∙ 4 H_2_O, 1.87 g L^−1^ ZnSO_4_∙ 7 H_2_O, 0.2 g L^−1^ H_3_BO_3_, 0.25 g L^−1^ Na_2_MoO_4_∙ 2 H_2_O, 0.15 g L^−1^ CuCl_2_∙ 2 H_2_O, 0.4 g L^−1^ Na_2_EDTA ∙ 2 H_2_O | (Bühler et al., 2003) |
| SOC-medium | 20 g L^−1^ tryptone, 5 g L^−1^ yeast extract, 0.584 g L^−1^ NaCl, 0.186 g L^−1^ KCl, 2.003 g L^−1^ MgCl_2_∙ 6 H_2_O, 10 mL L^−1^ MgSO_4_, 7.2 mL L^−1^ 50 % (w/v) glucose | (Hanahan, 1983) |
| Phosphate-buffered saline (PBS) | 8 g L^−1^NaCl, 0.2 g L^−1^KCl, 1.44 g L^−1^Na_2_HPO_4_, 0.24 g L^−1^KH_2_PO_4_, pH 7.4 | (Sambrook and Russell, 2001) |

**Table S2: Strains.** *E. coli* DH5α λpir was used for cloning and propagation of pEMG plasmids. *E. coli* DH5α was used for cloning and propagation of all other plasmids.

| Strain | Description | Reference |
| --- | --- | --- |
| *E. coli* |  |  |
| DH5α | *sup*E44 Δ*lacU*169 (φ80 *lac*ZΔM15) *hsd*R17 *rec*A1 *end*A1 *gyr*A96 *thi*-1 *rel*A1 | (Hanahan, 1983) |
| DH5α λpir | λpir lysogen of DH5a | (Martínez-García and de Lorenzo, 2011) |
| *Pseudomonas putida* |  |  |
| KT2440 | wild-type strain | (Bagdasarian et al., 1981) |
| *Pseudomonas taiwanensis* |  |  |
| VLB120ΔC | VLB120 with disruption of *styC* on the megaplasmid pSTY by insertion of Sm^R^ | (Park et al., 2007) |
| VLB120ΔC pCom10lac | VLB120ΔC with the pCom10lac plasmid, Sm^R^, Km^R^ | This work |
| VLB120ΔC pCom10lac_*kguT* | VLB120ΔC with the pCom10lac plasmid harboring *kguT*, Sm^R^, Km^R^ | This work |
| VLB120ΔC pCom10lac_*gntP* | VLB120ΔC with the pCom10lac plasmid harboring *gntP*, Sm^R^, Km^R^ | This work |
| VLB120ΔC pCom10lac_PVLB18545 | VLB120ΔC with the pCom10lac plasmid harboring PVLB_18545, Sm ^R^, Km^R^ | This work |
| VLB120ΔC pCom10Syn35T | VLB120ΔC with the pCom10Syn35T plasmid, Sm^R^, Km^R^ | This work |
| VLB120ΔC pCom10Syn35T_PVLB12345 | VLB120ΔC with the pCom10Syn35T plasmid harboring PVLB_12345, Sm^R^, Km^R^ | This work |
| VLB120ΔC pCom10Syn35T_PVLB05820 | VLB120ΔC with the pCom10Syn35T plasmid harboring PVLB_05820, Sm^R^, Km^R^ | This work |
| VLB120ΔCΔ*gntR* | VLB120ΔC with deletion of *gntR,* Sm^R^ | This work |
| VLB120ΔCΔ*gntR* pCom10Syn35T | VLB120ΔCΔ*gntR* with the pCom10Syn35T (empty vector) plasmid, Sm^R^, Km^R^ | This work |
| VLB120ΔC*gntR* pCom10Syn35T_PVLB12345 | VLB120ΔC*gntR* with the pCom10Syn35T plasmid harboring PVLB_12345, Sm^R^, Km^R^ | This work |
| VLB120ΔC*gntR* pCom10Syn35T_PVLB05820 | VLB120ΔC*gntR* with the pCom10Syn35T plasmid harboring PVLB_05820, Sm^R^, Km^R^ | This work |
| VLB120ΔCΔ*gntP* | VLB120ΔC with a deletion of *gntP,* Sm^R^ | This work |
| VLB120ΔCΔPVLB_18545 | VLB120ΔC with deletion of PVLB_18545*,* Sm^R^ | This work |
| VLB120ΔCΔ*gntP*ΔPVLB_18545 | VLB120ΔC with deletion of *gntP* and PVLB_18545*,* Sm^R^ | This work |
| VLB120ΔCΔ*gntP*ΔPVLB_18545 pCom10lac | VLB120ΔCΔ*gntP*ΔPVLB_18545 harboring the pCom10lac plasmid, Sm^R^, Km^R^ | This work |
| VLB120ΔCΔ*gntP*ΔPVLB18545 pCom10lac_*kguT* | VLB120ΔCΔ*gntP*ΔPVLB_18545 with the pCom10lac_*kguT* plasmid harboring *kguT,* Sm^R^, Km^R^ | This work |
| VLB120ΔCΔ*gntP*ΔPVLB18545 pCom10lac_*gntP* | VLB120ΔCΔ*gntP*ΔPVLB_18545 with the pCom10lac_*kguT* plasmid harboring *gntP*, Sm^R^, Km^R^ | This work |
| VLB120ΔCΔ*gntP*ΔPVLB18545 pCom10lac_PVLB18545 | VLB120ΔCΔ*gntP*ΔPVLB_18545PVLB with the pCom10lac_*kguT* plasmid harboring PVLB_18545, Sm^R^, Km^R^ | This work |

## Table S3: Plasmids.

| Plasmid | Description | Reference |
| --- | --- | --- |
| pCom10lac | ColE1 and pRO1600 ori, *lac*-regulatory system (*lac*I, P_lacUV5_), Km^R^ (empty vector) | (Lindmeyer, 2016) |
| pCom10lac_*kguT* | pCom10lac with *kguT* from *P. putida* KT2440 | This work |
| pCom10lac_*gntP* | pCom10lac with *gntP* from *P. taiwanensis* VLB120 | This work |
| pCom10lac_PVLB18545 | pCom10lac with PVLB_18545 from *P. taiwanensis* VLB120 | This work |
| pCom10Syn35T | ColE1 and pRO1600 ori, synthetic constitutive promoter Syn35T, Km^R^ (empty vector) | This work |
| pCom10Syn35T_PVLB12345 | pCom10Syn35T with PVLB_12345 from *P. taiwanensis* VLB120 | This work |
| pCom10Syn35T_PVLB05820 | pCom10Syn35T with PVLB_05820 from *P. taiwanensis* VLB120 | This work |
| pEMG | R6K ori, *lac*Za with two flanking I-SceI sites, Km^R^ | (Martínez-García and de Lorenzo, 2011) |
| pEMG_*ttgV* | pEMG harboring a ~ 1 kb EcoRI-XbaI fragment for deletion of *ttgV*, Km^R^ | (Volmer et al., 2014) |
| pEMG_PVLB13665 | pEMG harboring a ~ 1 kb EcoRI-XbaI fragment for deletion of PVLB_13665, Km^R^ | This work |
| pEMG_PVLB18545 | pEMG harboring a ~ 1 kb EcoRI-XbaI fragment for deletion of PVLB_18545, Km^R^ | This work |
| pEMG_PVLB13655 | pEMG harboring a ~ 1 kb EcoRI-XbaI fragment for deletion of PVLB_13655, Km^R^ | This work |
| pSW-2 | RK2 ori, *xylS*, *Pm* promoter, I-SceI gene, Gm^R^ | (Martínez-García and de Lorenzo, 2011) |

Table S4: Primers. FW (forward), RV (reverse), HR (homology region).

| Primer | Sequence (5‘ → 3‘) | Description |
| --- | --- | --- |
| PPN103 | AGGAGGTTTTCTAagccaacctgGGGTTGGCTTTTTTATGCAataatgcagcctgaaaggcag | FW primer for linearization and elimination of lac promoter system from pCom10lac; adds overhang for Syn35T cassette |
| PPN104 | AAGGTTGAAAAATAAAAACGGCGCTAAAAAGCGCCGTTTTTTTTGACGGTGGTAttaggcaccgggatctcgac | RV primer for linearization and elimination of lac promoter system from pCom10lac; adds overhang for Syn35T cassette |
| PPN105 | AGCGCCGTTTTTATTTTTCAACCTTCGCATACGCTACTTGCATTACAGTTTACGAACCGAACAGGCTTATGTCAAGACGTCTTAATTAA | FW primer for addition of Syn35T promoter cassette by "primer only PCR" with PPN 106; overhang with PCR product of PPN103/104 |
| PPN106 | CCcaggttggctTAGAAAACCTCCTCCTAGGCCCCAAATTATAATTCTAAACATCACGCATGTCAAATAAATTAATTAAGACGTCTTGACATAAGC | RV primer for addition of Syn35T promoter cassette by "primer only PCR" with PPN 105; overhang with PCR product of PPN103/104 |
| PPN107 | agccaacctgGGGTTGG | FW primer for amplification of whole plasmid pCom10_Syn35T for Gibson cloning |
| PPN108 | TAGAAAACCTCCTCCTAGGCCC | RV primer for amplification of whole plasmid pCom10_Syn35T for Gibson cloning |
| PPN116 | CCTAGGAGGAGGTTTTCTAatgaactgcgaactgatcgtcgacg | FW primer for amplification of PVLB_05820 for Gibson cloning |
| PPN117 | AACCCcaggttggcttcagccccggtaggcaggtt | RV primer for amplification of PVLB_05820 for Gibson cloning |
| PPN118 | CCTAGGAGGAGGTTTTCTAatgccgttcacagacccat | FW primer for amplification of PVLB_12345 for Gibson cloning |
| PPN119 | AACCCcaggttggcttcattccgtgaacgtgaaacgac | RV primer for amplification of PVLB_12345 for Gibson cloning |
| PPN132 | AGGGTAATCTGAATTCTGGCAGGCATGAAGTGGC | Gene knockout of PVLB_13655: FW primer of HR1 |
| PPN133 | CGCACTCGCGTAGCGCTGTCTCAGGACG | Gene knockout of PVLB_13655: RV primer of HR1 with 5' overhang for HR2 |
| PPN134 | CAGCGCTACGCGAGTGCGTAATCAGGCTT | Gene knockout of PVLB_13655: FW primer of HR2 with 5' overhang for HR1 |
| PPN135 | GCAGGTCGACTCTAGACTGATATCAGCGGCGACCTT | Gene knockout of PVLB_13655: RV primer of HR2 |
| PPN136 | AGGGTAATCTGAATTCTCTGAACGACGACGACCG | Gene knockout of PVLB_13665: FW primer of HR1 |
| PPN137 | ACCACCAACATGCCTCGGTCTTATCGTTGT | Gene knockout of PVLB_13665: RV primer of HR1 with 5' overhang for HR2 |
| PPN138 | CCGAGGCATGTTGGTGGTGTGACCCTCAAG | Gene knockout of PVLB_13665: FW primer of HR2 with 5' overhang of HR1 |
| PPN139 | GCAGGTCGACTCTAGACCTTGAAGGTGCCACGTCT | Gene knockout of PVLB_13665: RV primer of HR2 |
| PPN170 | AGGGTACTGAATTCGTGGTTGAACTGGCTGACCAG | Gene knockout of PVLB_18545: FW primer of HR1 |
| PPN171 | TCTCCGGCTCGACATTGTGTTGAGCCATGGC | Gene knockout of PVLB_18545: RV primer of HR1 with 5' overhang for HR2 |
| PPN172 | ACACAATGTCGAGCCGGAGATGGCATGAG | Gene knockout of PVLB_18545: FW primer of HR2 with 5' overhang for HR1 |
| PPN173 | GCAGGTCGACTCTAGAacgtcTCATCCTCAATCGGCTGTTCGAG | Gene knockout of PVLB_18545: RV primer of HR2 |
| PPN182 | ccagtactggagaattccatATGCAAAGCCAAAGCCTGG | FW primer for amplification of PP_3377 (*kguT*) for cloning into pCom10lac |
| PPN183 | cgggcgcgccaagcaCTAATGGTTCATGGCCAGACG | RV primer for amplification of PP_3377 (*kguT*) for cloning into pCom10lac |
| PPN184 | ccagtactggagaattccatATGGCTCAACACAATGTCGCC | FW primer for amplification of PVLB_18545 for cloning into pCom10lac |
| PPN185 | cgggcgcgccaagcaTCATGCCATCTCCGGCTCC | RV primer for amplification of PVLB_18545 for cloning into pCom10lac |
| PPN186 | ccagtactggagaattccatATGTTCGGACTGGCAACTGATAC | FW primer for amplification of PVLB_13665 for cloning into pCom10lac |
| PPN187 | cgggcgcgccaagcaTCACACCACCAACGACAGC | RV Gibson primer for cloning PVLB_13665 for cloning into pCom10lac |
| SPPN001 | CGGTCGATCATTCAGCCCG | FW primer for sequencing/colony PCR of pCom10lac insert |
| SPPN002 | TGCCGCCAGGCAAATTCTG | RV primer for sequencing/colony PCR of pCom10lac insert |
| SPPN027 | CAGGCTGCGCAACTGTTG | FW primer for sequencing/colony PCR of pEMG insert |
| SPPN028 | GCGTTGGCCGATTCATTAATGC | RV primer for sequencing/colony PCR of pEMG insert |
| SPPN039 | CGAACAGGCTTATGTCAAGACGTCTTAATTA | FW primer for sequencing/colony PCR of pCom10_Syn35T insert |
| SPPN040 | CCTGCCTTTCAGGCTGCATTA | RV primer for sequencing/colony PCR of pCom10_Syn35T insert |

**
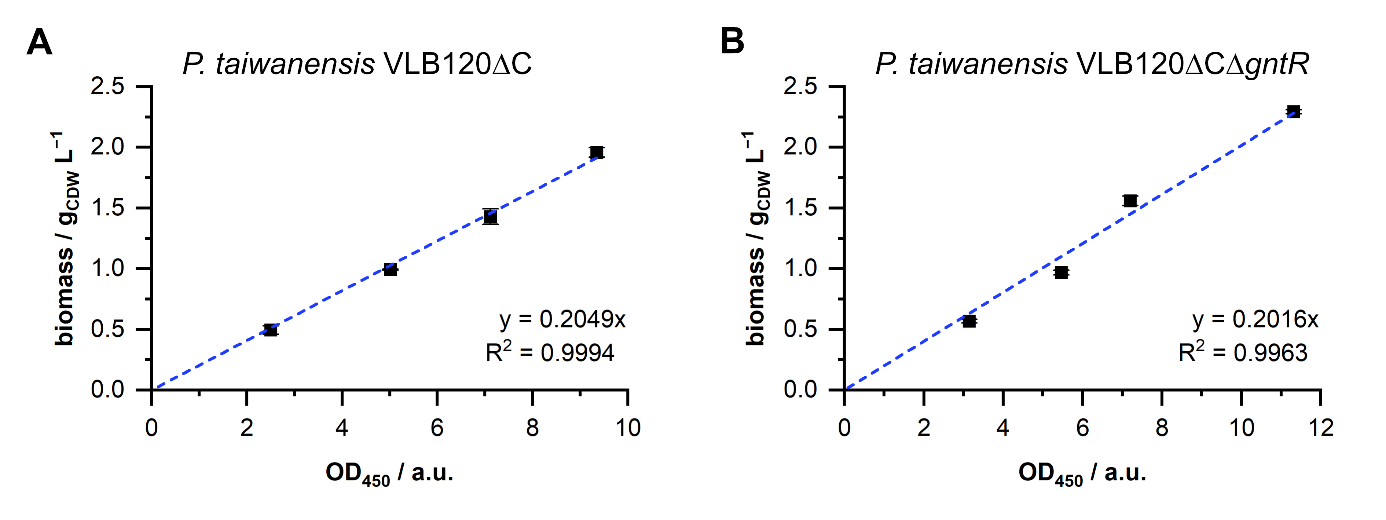
**

Figure S1: Correlation of optical density at 450 nm (OD_450_) and biomass concentration cell dry weight (CDW). For the exact procedure, see the materials and methods section.

**
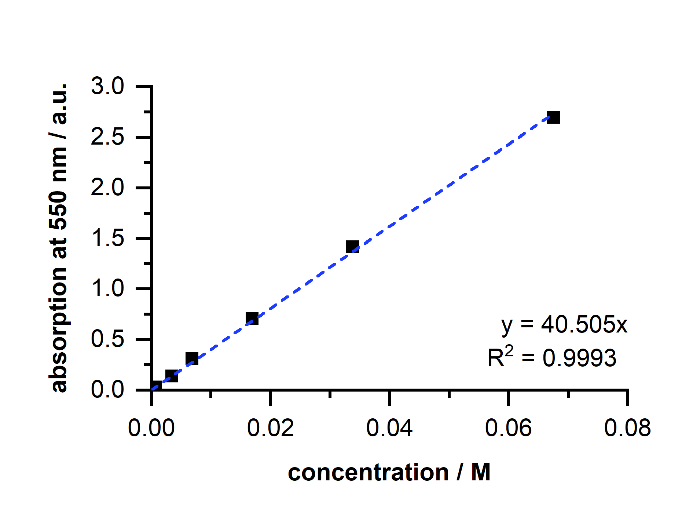
**

## Figure S2: Correlation between absorption at 550 nm and d-xylonolactone concentration (hydroxamate assay).

**
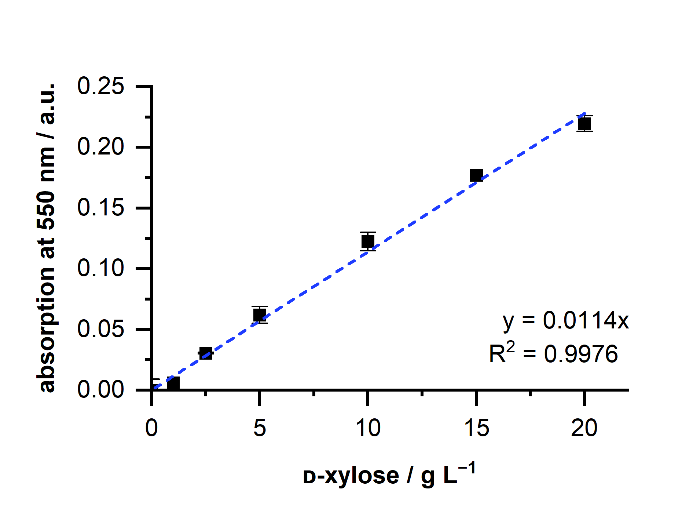
**

## Figure S3: Correlation between absorption at 550 nm and d-xylose concentration (hydroxamate assay).

**
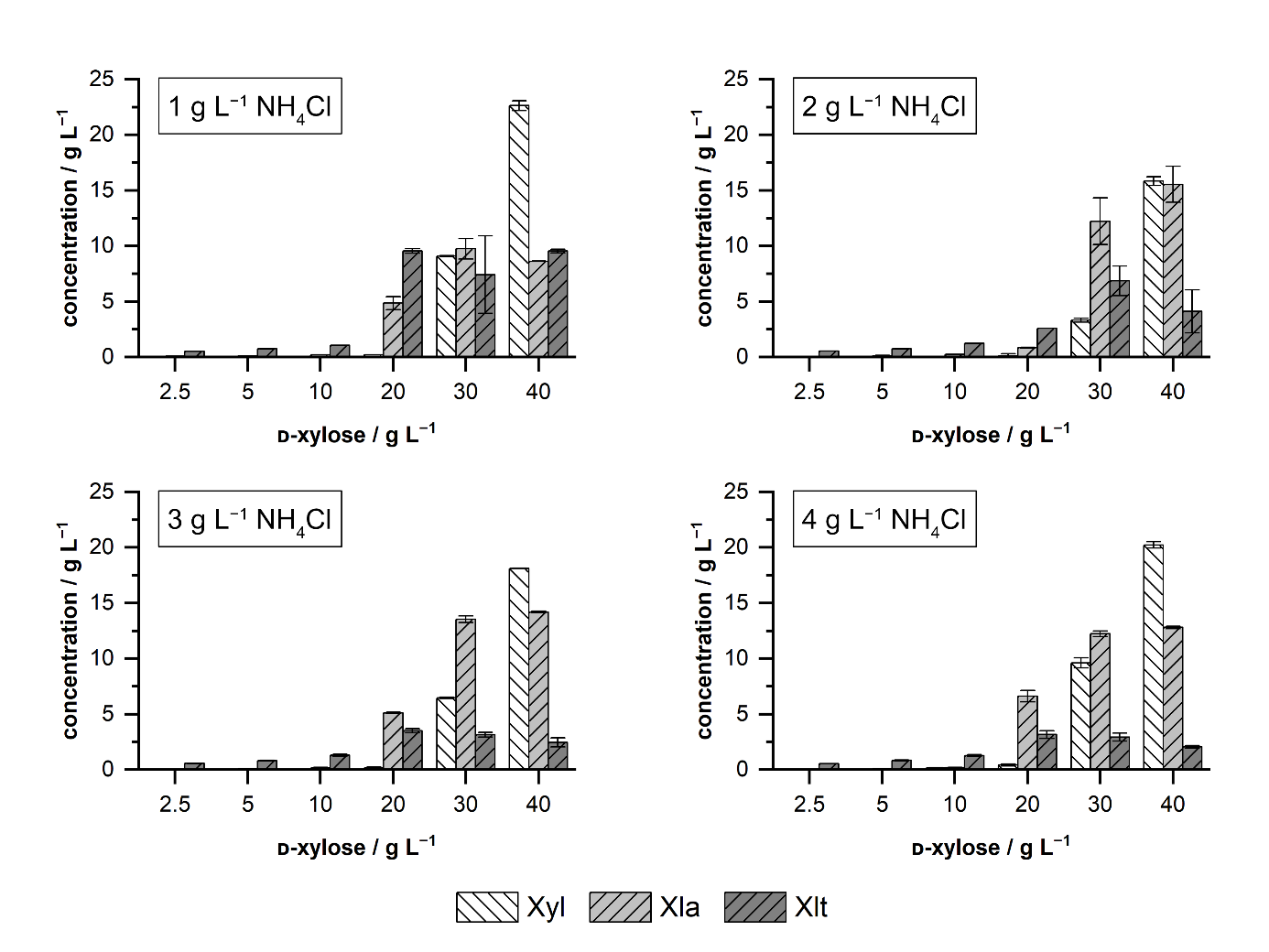
**

Figure S4: Final extracellular substrate and metabolite concentrations of BioLector cultivations with different concentrations of d-xylose and ammonium chloride (NH_4_Cl). Depicted are the concentrations of d-xylose (Xyl), d-xylonolactone (Xla) and d-xylonate (Xlt) at the end of the cultivations. Cultivations were performed in a BioLector I in M9 medium at 1 mL scale, 1,200 rpm, 30 °C for 72 h. Mean values and error bars (standard deviation) are calculated from two biologically independent cultivations.


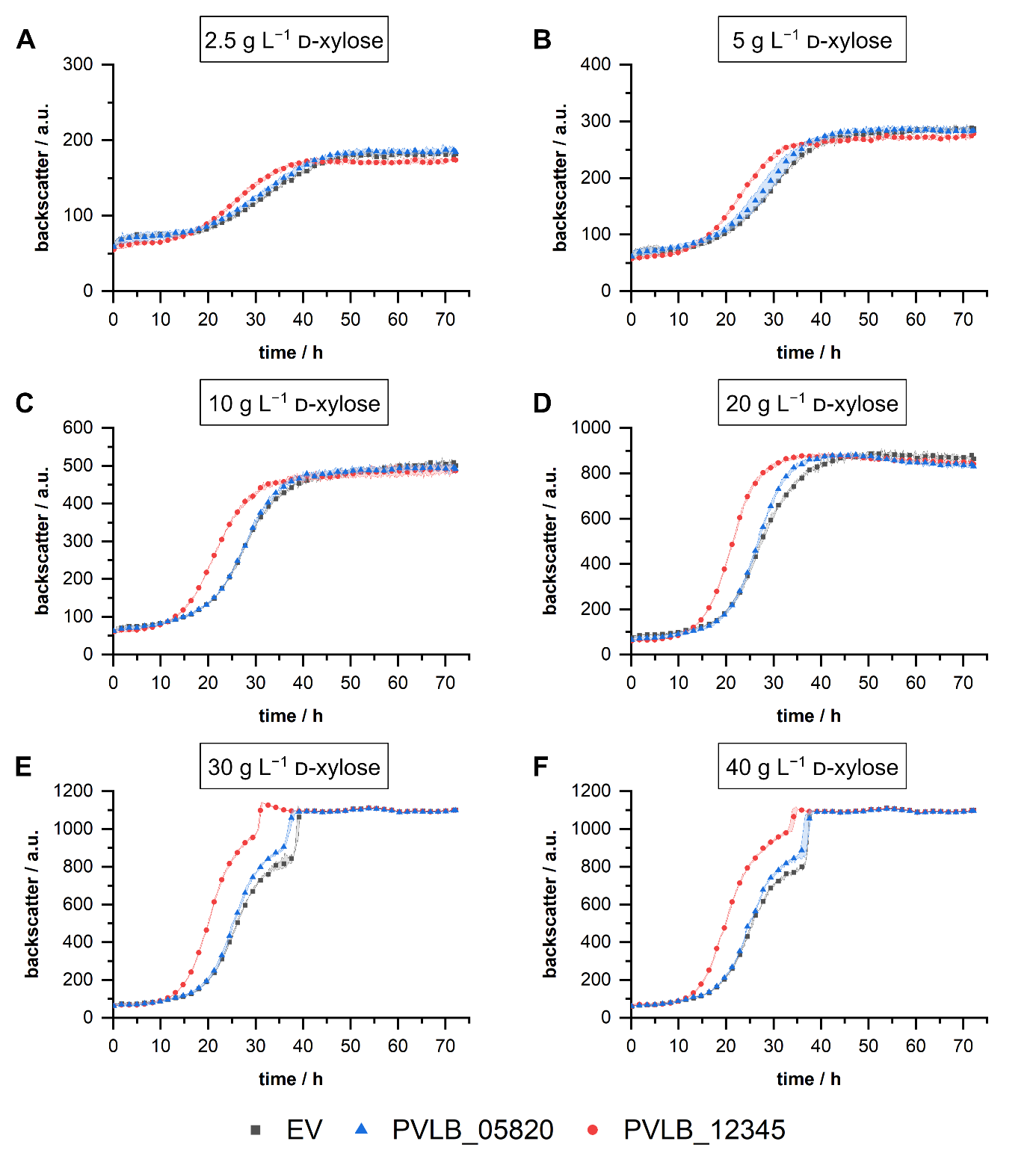


Figure S5: BioLector growth curves of cultivations with plasmid-based overexpression of putative lactonase genes. Cultivations of the empty vector control (EV) and the strains overexpressing PVLB_05820 and PVLB_12345 were performed in M9 medium (2 g L^−1^ NH_4_Cl) with 2.5 g L^−1^ **(A)**, 5 g L^−1^ **(B)**, 10 g L^−1^ **(C)**, 20  g L^−1^ **(D)**, 30 g L^−1^ **(E)** or 40  g L^−1^ of d-xylose **(F)** were performed in a BioLector I at 1 mL scale, 1,200 rpm, 30 °C for 72 h. Every eighth mean value of at least two independent biological cultivations is depicted. The standard deviations for all measurements are shown as error bands. The sharp increase in backscatter in E and F after 30 – 40 h of cultivation was a result of intense foam formation.

Table S5: Protein BLAST search for the amino acid sequence of KguT in the genome of *P. taiwanensis* VLB120. All corresponding proteins are annotated as belonging to the major facilitator superfamily (MFS).

| Gene | Max Score | Query Cover | E-value | Percent Identity |
| --- | --- | --- | --- | --- |
| PVLB_18545 | 137 | 87 % | 7e−37 | 30.18 % |
| PVLB_03325 | 132 | 97 % | 6e−35 | 28.44 % |
| PVLB_12280 | 129 | 88 % | 7e−34 | 29.85 % |
| PVLB_12720 | 67.8 | 86 % | 5e−13 | 23.00 % |

**
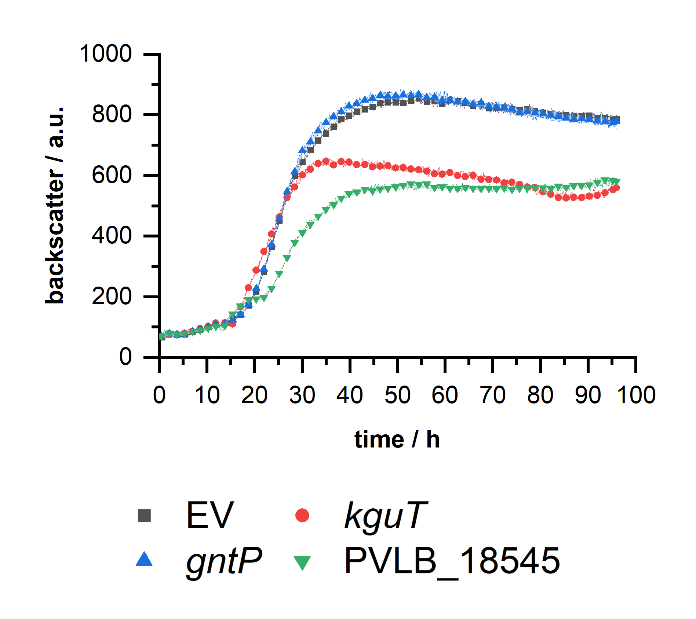
**

Figure S6: BioLector growth curves of cultivations with plasmid-based overexpression of xylonate transporter genes. Cultivations of the empty vector control (EV) and the strains overexpressing *gntP*, PVLB_18545, and *kguT* were performed in a BioLector I in M9 medium (20 g L^−1^ d-xylose and 2 g L^−1^ NH_4_Cl) at 1 mL scale, 1,200 rpm, 30 °C for 96 h. Every eighth mean value of two independent biological cultivations is depicted. The standard deviations for all measurements are shown as error bands.

**
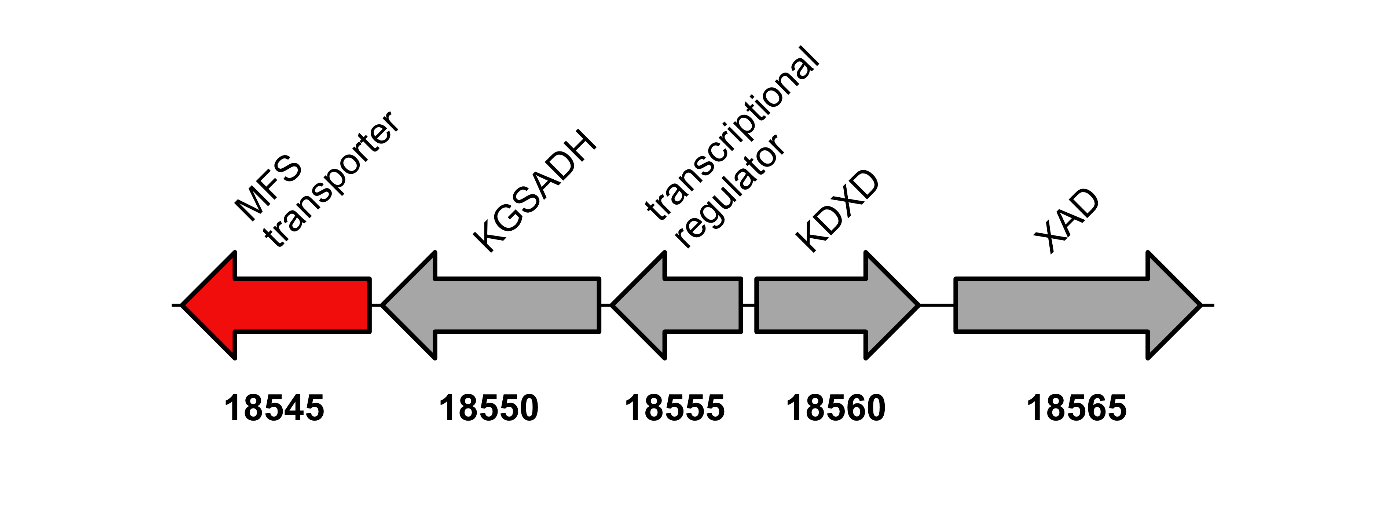
**Figure S7: Genetic arrangement of the Weimberg pathway genes of *P. taiwanensis* VLB120. Xylonate dehydratase (XAD), 2-keto-3-deoxy-d-xylonate dehydratase (KDXD), α-ketoglutarate semialdehyde dehydrogenase (KGSADH), major facilitator superfamily transporter (MFS transporter). The numbers in the figure represent the locus tags without PVLB prefix.


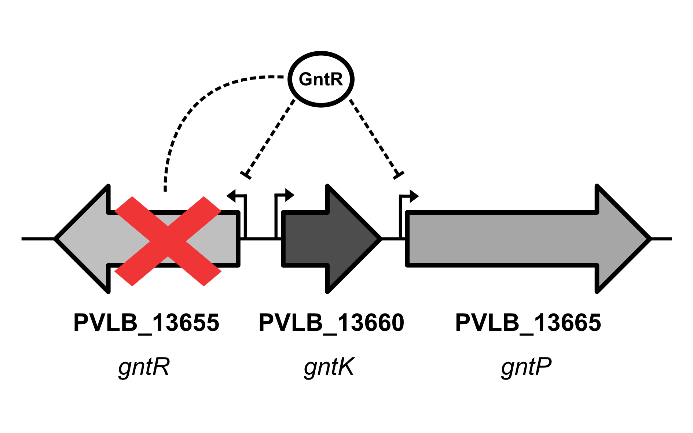


**Figure S8: Deregulation of GntP in *P. taiwanensis* VLB120ΔC.** Schematic presentation of the gene cluster consisting of *gntR* (PVLB_13655), *gntK* (PVLB_13660) and *gntP* (PVLB_13665) coding for a transcriptional regulator, gluconokinase and gluconate permease, respectively.

Table S6: Post hoc analysis data for the growth rates of Figure 4. The F-test of the one-way ANOVA resulted in a significant difference in the group means at the level of 0.05. Bonferroni post hoc analysis was performed to explore significant difference between means.

|  | Mean Difference | SEM | t-value | p-value | Alpha | Sig |
| --- | --- | --- | --- | --- | --- | --- |
| **2.5 g L^−1^ d-xylose** | | | | | | |
| PVLB_05820 EV | 6.56E-04 | 6.41E-04 | 1 | 1 | 0.05 | 0 |
| PVLB_12345 EV | 0.01061 | 6.41E-04 | 17 | <0.0001 | 0.05 | 1 |
| PVLB_12345 PVLB_05820 | 0.00996 | 5.73E-04 | 17 | <0.0001 | 0.05 | 1 |
| **5 g L^−1^ d-xylose** |  |  |  |  |  |  |
| PVLB_05820 EV | 0.00232 | 0.00311 | 0.74612 | 1 | 0.05 | 0 |
| PVLB_12345 EV | 0.01885 | 0.00311 | 6 | 0.00529 | 0.05 | 1 |
| PVLB_12345 PVLB_05820 | 0.01653 | 0.00278 | 6 | 0.00578 | 0.05 | 1 |
| **10 g L^−1^ d-xylose** |  |  |  |  |  |  |
| PVLB_05820 EV | -5.13E-04 | 0.00139 | -0.36769 | 1 | 0.05 | 0 |
| PVLB_12345 EV | 0.01749 | 0.00139 | 13 | 1.72E-04 | 0.05 | 1 |
| PVLB_12345 PVLB_05820 | 0.018 | 0.00125 | 14 | <0.0001 | 0.05 | 1 |
| **20 g L^−1^ d-xylose** |  |  |  |  |  |  |
| PVLB_05820 EV | 0.01249 | 0.00195 | 6 | 0.00414 | 0.05 | 1 |
| PVLB_12345 EV | 0.03916 | 0.00195 | 20 | <0.0001 | 0.05 | 1 |
| PVLB_12345 PVLB_05820 | 0.02667 | 0.00175 | 15 | <0.0001 | 0.05 | 1 |
| **30 g L^−1^ d-xylose** |  |  |  |  |  |  |
| PVLB_05820 EV | 0.01267 | 0.00197 | 6 | 0.00404 | 0.05 | 1 |
| PVLB_12345 EV | 0.04462 | 0.00197 | 23 | <0.0001 | 0.05 | 1 |
| PVLB_12345 PVLB_05820 | 0.03195 | 0.00176 | 18 | <0.0001 | 0.05 | 1 |
| **40 g L^−1^ d-xylose** |  |  |  |  |  |  |
| PVLB_05820 EV | 0.00972 | 0.00193 | 5 | 0.01189 | 0.05 | 1 |
| PVLB_12345 EV | 0.04511 | 0.00193 | 23 | <0.0001 | 0.05 | 1 |
| PVLB_12345 PVLB_05820 | 0.03539 | 0.00172 | 21 | <0.0001 | 0.05 | 1 |

Table S7: Post hoc analysis data for the biomass concentrations of Figure 4. The F-test of the one-way ANOVA resulted in a significant difference in the group means at the level of 0.05. Bonferroni post hoc analysis was performed to explore significant difference between means.

|  | Mean Difference | SEM | t-value | p-value | Alpha | Sig |
| --- | --- | --- | --- | --- | --- | --- |
| **2.5 g L^−1^ d-xylose** | | | | | | |
| PVLB_05820 EV | -3.07E-03 | 9.99E-03 | 0 | 1 | 0.05 | 0 |
| PVLB_12345 EV | 0.042 | 9.99E-03 | 4 | 0.02531 | 0.05 | 1 |
| PVLB_12345 PVLB_05820 | 0.04508 | 8.93E-03 | 5 | 0.01183 | 0.05 | 1 |
| **5 g L^−1^ d-xylose** |  |  |  |  |  |  |
| PVLB_05820 EV | 0.08537 | 0.02623 | 3.25472 | 0.06773 | 0.05 | 0 |
| PVLB_12345 EV | 0.2288 | 0.02623 | 9 | 9.84E-04 | 0.05 | 1 |
| PVLB_12345 PVLB_05820 | 0.14343 | 0.02346 | 6 | 0.00509 | 0.05 | 1 |
| **10 g L^−1^ d-xylose** |  |  |  |  |  |  |
| PVLB_05820 EV | -3.76E-02 | 0.10032 | -0.37444 | 1 | 0.05 | 0 |
| PVLB_12345 EV | 0.31077 | 0.10032 | 3 | 8.08E-02 | 0.05 | 0 |
| PVLB_12345 PVLB_05820 | 0.34833 | 0.08973 | 4 | 0.03486 | 0.05 | 1 |
| **20 g L^−1^ d-xylose** |  |  |  |  |  |  |
| PVLB_05820 EV | -0.24588 | 0.07482 | -3 | 0.0654 | 0.05 | 0 |
| PVLB_12345 EV | 0.4098 | 0.07482 | 5 | 0.00829 | 0.05 | 1 |
| PVLB_12345 PVLB_05820 | 0.65568 | 0.06692 | 10 | 5.66E-04 | 0.05 | 1 |
| **30 g L^−1^ d-xylose** |  |  |  |  |  |  |
| PVLB_05820 EV | 0.31077 | 0.56213 | 1 | 1 | 0.05 | 0 |
| PVLB_12345 EV | 1.74507 | 0.56213 | 3 | 0.08016 | 0.05 | 0 |
| PVLB_12345 PVLB_05820 | 1.4343 | 0.50278 | 3 | 0.10713 | 0.05 | 0 |
| **40 g L^−1^ d-xylose** |  |  |  |  |  |  |
| PVLB_05820 EV | 0.30394 | 0.70494 | 0 | 1 | 0.05 | 0 |
| PVLB_12345 EV | 2.98813 | 0.70494 | 4 | 0.02454 | 0.05 | 1 |
| PVLB_12345 PVLB_05820 | 2.68419 | 0.63052 | 4 | 0.02411 | 0.05 | 1 |

Table S8: Post hoc analysis data for the pH values of Figure 4. The F-test of the one-way ANOVA resulted in a significant difference in the group means at the level of 0.05. Bonferroni post hoc analysis was performed to explore significant difference between means.

|  | Mean Difference | SEM | t-value | p-value | Alpha | Sig |
| --- | --- | --- | --- | --- | --- | --- |
| **2.5 g L^−1^ d-xylose** | | | | | | |
| PVLB_05820 EV | -1.67E-02 | 3.33E-03 | -5 | 0.01231 | 0.05 | 1 |
| PVLB_12345 EV | 0 | 3.33E-03 | 0 | 1 | 0.05 | 0 |
| PVLB_12345 PVLB_05820 | 0.01667 | 2.98E-03 | 6 | 0.00758 | 0.05 | 1 |
| **5 g L^−1^ d-xylose** |  |  |  |  |  |  |
| PVLB_05820 EV | -0.00167 | 0.00553 | -0.30151 | 1 | 0.05 | 0 |
| PVLB_12345 EV | 0.01833 | 0.00553 | 3 | 6.33E-02 | 0.05 | 0 |
| PVLB_12345 PVLB_05820 | 0.02 | 0.00494 | 4 | 0.02962 | 0.05 | 1 |
| **10 g L^−1^ d-xylose** |  |  |  |  |  |  |
| PVLB_05820 EV | 3.33E-02 | 0.00667 | 5 | 0.01231 | 0.05 | 1 |
| PVLB_12345 EV | 0.06 | 0.00667 | 9 | 8.48E-04 | 0.05 | 1 |
| PVLB_12345 PVLB_05820 | 0.02667 | 0.00596 | 4 | 0.0197 | 0.05 | 1 |
| **20 g L^−1^ d-xylose** |  |  |  |  |  |  |
| PVLB_05820 EV | 0.04833 | 0.0128 | 4 | 0.03885 | 0.05 | 1 |
| PVLB_12345 EV | 0.23167 | 0.0128 | 18 | <0.0001 | 0.05 | 1 |
| PVLB_12345 PVLB_05820 | 0.18333 | 0.01145 | 16 | <0.0001 | 0.05 | 1 |
| **30 g L^−1^ d-xylose** |  |  |  |  |  |  |
| PVLB_05820 EV | 1.06167 | 0.4793 | 2 | 0.23284 | 0.05 | 0 |
| PVLB_12345 EV | 0.18833 | 0.4793 | 0 | 1 | 0.05 | 0 |
| PVLB_12345 PVLB_05820 | -0.87333 | 0.4287 | -2 | 0.29166 | 0.05 | 0 |
| **40 g L^−1^ d-xylose** |  |  |  |  |  |  |
| PVLB_05820 EV | 0.46167 | 0.03738 | 12 | 1.85E-04 | 0.05 | 1 |
| PVLB_12345 EV | 0.37833 | 0.03738 | 10 | 4.84E-04 | 0.05 | 1 |
| PVLB_12345 PVLB_05820 | -0.08333 | 0.03343 | -2 | 0.16497 | 0.05 | 0 |

Table S9: Post hoc analysis data for the concentrations of d-xylonolactone and d-xylonate of Figure 4. The F-test of the one-way ANOVA resulted in a significant difference in the group means at the level of 0.05. Bonferroni post hoc analysis was performed to explore significant difference between means.

|  | Mean Difference | | SEM | t-value | p-value | Alpha | Sig |
| --- | --- | --- | --- | --- | --- | --- | --- |
| **30 g L^−1^ d-xylose: d-xylonolactone** | | | | | | | |
| PVLB_05820 EV | | -9.34E+00 | 1.61E-01 | -58 | <0.0001 | 0.05 | 1 |
| PVLB_12345 EV | | -6.63282 | 1.61E-01 | -41 | <0.0001 | 0.05 | 1 |
| PVLB_12345 PVLB_05820 | | 2.70429 | 1.44E-01 | 19 | <0.0001 | 0.05 | 1 |
| **30 g L^−1^ d-xylose: d-xylonate** | | | | | | | |
| PVLB_05820 EV | | 1.97354 | 1.19511 | 1.65134 | 0.47874 | 0.05 | 0 |
| PVLB_12345 EV | | 3.33138 | 1.19511 | 3 | 1.16E-01 | 0.05 | 0 |
| PVLB_12345 PVLB_05820 | | 1.35784 | 1.06894 | 1 | 0.77967 | 0.05 | 0 |
| **40 g L^−1^ d-xylose: d-xylonolactone** | | | | | | | |
| PVLB_05820 EV | | -9.29E+00 | 0.30297 | -30.66396 | <0.0001 | 0.05 | 1 |
| PVLB_12345 EV | | -8.66156 | 0.30297 | -29 | <0.0001 | 0.05 | 1 |
| PVLB_12345 PVLB_05820 | | 0.62865 | 0.27098 | 2 | 0.20419 | 0.05 | 0 |
| **40 g L^−1^ d-xylose: d-xylonate** | | | | | | | |
| PVLB_05820 EV | | 3.92372 | 0.56268 | 7 | 0.0028 | 0.05 | 1 |
| PVLB_12345 EV | | 4.07489 | 0.56268 | 7 | 0.00235 | 0.05 | 1 |
| PVLB_12345 PVLB_05820 | | 0.15118 | 0.50327 | 0 | 1.00E+00 | 0.05 | 0 |

Table S10: Post hoc analysis data for the mean values presented in Figure 6. The F-test of the one-way ANOVA resulted in a significant difference in the group means at the level of 0.05. Bonferroni post hoc analysis was performed to explore significant difference between means.

|  | Mean Difference | SEM | t-value | p-value | Alpha | Sig |
| --- | --- | --- | --- | --- | --- | --- |
| **Growth rate** | | | | | | |
| kguT EV | 0.08916 | 0.00268 | 33.30875 | <0.0001 | 0.05 | 1 |
| gntP EV | 0.00109 | 0.00268 | 0.4074 | 1 | 0.05 | 0 |
| gntP kguT | -0.08807 | 0.00268 | -32.90135 | <0.0001 | 0.05 | 1 |
| PVLB_18545 EV | 0.05579 | 0.00268 | 20.84355 | 1.88E-04 | 0.05 | 1 |
| PVLB_18545 kguT | -0.03337 | 0.00268 | -12.4652 | 0.00143 | 0.05 | 1 |
| PVLB_18545 gntP | 0.0547 | 0.00268 | 20.43615 | 2.03E-04 | 0.05 | 1 |
| **Biomass** | | | | | | |
| kguT EV | -1.86459 | 0.0575 | -32.4277 | <0.0001 | 0.05 | 1 |
| gntP EV | 0.24588 | 0.0575 | 4.27618 | 0.07732 | 0.05 | 0 |
| gntP kguT | 2.11047 | 0.0575 | 36.70388 | <0.0001 | 0.05 | 1 |
| PVLB_18545 EV | -1.1372 | 0.0575 | -19.77733 | 2.31E-04 | 0.05 | 1 |
| PVLB_18545 kguT | 0.72739 | 0.0575 | 12.65037 | 0.00135 | 0.05 | 1 |
| PVLB_18545 gntP | -1.38308 | 0.0575 | -24.05351 | 1.06E-04 | 0.05 | 1 |
| **d-Xylonolactone** | | | | | | |
| kguT EV | 0.06456 | 0.05551 | 1.16309 | 1 | 0.05 | 0 |
| gntP EV | 0.01107 | 0.05551 | 0.19939 | 1 | 0.05 | 0 |
| gntP kguT | -0.0535 | 0.05551 | -0.96371 | 1 | 0.05 | 0 |
| PVLB_18545 EV | -0.06459 | 0.05551 | -1.16354 | 1 | 0.05 | 0 |
| PVLB_18545 kguT | -0.12915 | 0.05551 | -2.32664 | 0.48329 | 0.05 | 0 |
| PVLB_18545 gntP | -0.07566 | 0.05551 | -1.36293 | 1 | 0.05 | 0 |
| **d-Xylonate** | | | | | | |
| kguT EV | -0.98926 | 0.0742 | -13.33285 | 0.0011 | 0.05 | 1 |
| gntP EV | 0.32701 | 0.0742 | 4.40732 | 0.06975 | 0.05 | 0 |
| gntP kguT | 1.31627 | 0.0742 | 17.74017 | 3.56E-04 | 0.05 | 1 |
| PVLB_18545 EV | -0.9728 | 0.0742 | -13.1111 | 0.00117 | 0.05 | 1 |
| PVLB_18545 kguT | 0.01645 | 0.0742 | 0.22175 | 1 | 0.05 | 0 |
| PVLB_18545 gntP | -1.29982 | 0.0742 | -17.51842 | 3.74E-04 | 0.05 | 1 |

Table S11: Biomass and d-xylose concentrations from stirred-tank bioreactor experiments presented in Figure 7. Colored values represent data for analysis of growth parameters.

| **VLB120ΔC pCom10Syn35T** | | | | **VLB120ΔC pCom10Syn35T_PVLB12345** | | | **VLB120ΔCΔgntR pCom10Syn35T** | | | | **VLB120ΔCΔgntR pCom10Syn35T_PVLB12345** | | | | |
| --- | --- | --- | --- | --- | --- | --- | --- | --- | --- | --- | --- | --- | --- | --- | --- |
| time / h | biomass  / g_CDW_ L^−1^ | d-xylose  / g L^−1^ | time / h | | biomass  / g_CDW_ L^−1^ | d-xylose  / g L^−1^ | | time / h | biomass  / g_CDW_ L^−1^ | d-xylose  / g L^−1^ | | time / h | biomass  / g_CDW_ L^−1^ | d-xylose  / g L^−1^ |  |
| 0.00 | 0.05 | 21.10 | 0.00 | | 0.05 | 20.94 | | 0.00 | 0.06 | 21.33 | | 0.00 | 0.06 | 21.54 |  |
| 3.05 | 0.08 | 20.87 | 3.00 | | 0.08 | 20.40 | | 2.98 | 0.09 | 20.82 | | 3.15 | 0.10 | 20.33 |  |
| 6.03 | 0.13 | 19.70 | 5.98 | | 0.14 | 20.40 | | 5.07 | 0.13 | 20.38 | | 5.97 | 0.18 | 19.73 |  |
| 8.15 | 0.17 | 19.63 | 8.10 | | 0.21 | 19.73 | | 6.92 | 0.20 | 19.29 | | 8.05 | 0.31 | 19.59 |  |
| 10.00 | 0.25 | 20.22 | 9.95 | | 0.31 | 20.15 | | 8.93 | 0.30 | 20.26 | | 9.95 | 0.50 | 19.38 |  |
| 11.98 | 0.35 | 19.08 | 11.93 | | 0.47 | 18.28 | | 15.52 | 1.48 | 13.40 | | 11.92 | 0.83 | 17.03 |  |
| 18.27 | 1.07 | 15.40 | 18.38 | | 2.05 | 11.84 | | 17.22 | 2.23 | 10.28 | | 18.50 | 4.14 | 5.16 |  |
| 20.08 | 1.58 | 13.33 | 20.12 | | 2.76 | 8.19 | | 19.15 | 2.85 | 6.14 | | 20.23 | 4.35 | 2.00 |  |
| 22.07 | 2.24 | 10.94 | 22.08 | | 3.74 | 4.43 | | 21.07 | 3.35 | 3.00 | | 22.15 | 5.37 | 0.50 |  |
| 24.02 | 2.98 | 6.86 | 24.08 | | 4.44 | 1.72 | | 23.13 | 4.04 | 1.00 | | 24.07 | 5.47 | 0.21 |  |
| 26.08 | 3.43 | 3.65 | 26.07 | | 5.50 | 0.51 | | 24.97 | 4.37 | 0.12 | | 26.12 | 5.61 | 0.02 |  |
| 27.98 | 3.95 | 2.23 | 27.95 | | 5.56 | 0.21 | | 26.97 | 4.86 | 0.33 | | 27.97 | 6.00 | 0.07 |  |
| 30.28 | 4.21 | 0.21 | 30.23 | | 5.91 | 0.00 | | 31.12 | 4.84 | 0.00 | | 30.22 | 6.40 | 0.00 |  |
| 34.10 | 4.32 | 0.42 | 34.07 | | 5.64 | 0.14 | | 32.98 | 5.14 | 0.00 | | 34.10 | 6.20 | 0.00 |  |
| 36.00 | 4.65 | 0.42 | 35.97 | | 6.13 | 0.00 | | 42.82 | 5.85 | 0.00 | | 35.95 | 6.10 | 0.00 |  |
| 45.83 | 5.51 | 0.00 | 45.80 | | 6.46 | 0.00 | | 45.02 | 5.48 | 0.00 | | 45.80 | 6.31 | 0.00 |  |
| 48.05 | 5.37 | 0.00 | 48.02 | | 6.16 | 0.00 | | 48.15 | 5.30 | 0.00 | | 48.00 | 6.03 | 0.00 |  |
| 58.83 | 5.62 | 0.00 | 58.80 | | 6.04 | 0.00 | | 55.78 | 5.63 | 0.00 | | 58.75 | 5.83 | 0.00 |  |
| 69.97 | 5.49 | 0.00 | 69.80 | | 5.62 | 0.00 | | 66.93 | 5.63 | 0.00 | | 69.90 | 5.62 | 0.00 |  |
| 72.13 | 5.43 | 0.00 | 72.10 | | 5.55 | 0.00 | | 72.00 | 5.59 | 0.00 | | 72.02 | 5.55 | 0.00 |  |

## Cited literature

Bagdasarian, M., Lurz, R., Rückert, B., Franklin, F.C.H., Bagdasarian, M.M., Frey, J., Timmis, K.N., 1981. Specific-purpose plasmid cloning vectors II. Broad host range, high copy number, RSF 1010-derived vectors, and a host-vector system for gene cloning in *Pseudomonas*. Gene 16, 237–247. https://doi.org/10.1016/0378-1119(81)90080-9

Bertani, G., 1951. Studies on lysogenesis. I. The mode of phage liberation by lysogenic *Escherichia coli*. J. Bacteriol. 62, 293–300. https://doi.org/10.1128/jb.62.3.293-300.1951

Bühler, B., Bollhalder, I., Hauer, B., Witholt, B., Schmid, A., 2003. Use of the two-liquid phase concept to exploit kinetically controlled multistep biocatalysis. Biotechnol. Bioeng. 81, 683–694. https://doi.org/10.1002/bit.10512

Hanahan, D., 1983. Studies on transformation of *Escherichia coli* with plasmids. J. Mol. Biol. 166, 557–580. https://doi.org/10.1016/S0022-2836(83)80284-8

Lindmeyer, M., 2016. *Pseudomonas* and heterogeneity – benefits and challenges for strain and process engineering, Chemical Biotechnology. Shaker Verlag, Aachen.

Martínez-García, E., de Lorenzo, V., 2011. Engineering multiple genomic deletions in Gram-negative bacteria: Analysis of the multi-resistant antibiotic profile of *Pseudomonas putida* KT2440. Environ. Microbiol. 13, 2702–2716. https://doi.org/10.1111/j.1462-2920.2011.02538.x

Park, J.-B., Bühler, B., Panke, S., Witholt, B., Schmid, A., 2007. Carbon metabolism and product inhibition determine the epoxidation efficiency of solvent-tolerant *Pseudomonas* sp. strain VLB120ΔC. Biotechnol. Bioeng. 98, 1219–1229. https://doi.org/10.1002/bit.21496

Sambrook, J., Russell, D.W., 2001. Molecular cloning: a laboratory manual, 3. ed. Cold Spring Harbor Laboratory Press, Cold Spring Harbor, NY.

Volmer, J., Neumann, C., Bühler, B., Schmid, A., 2014. Engineering of *Pseudomonas taiwanensis* VLB120 for constitutive solvent tolerance and increased specific styrene epoxidation activity. Appl. Environ. Microbiol. 80, 6539–6548. https://doi.org/10.1128/AEM.01940-14
